# Multivariate analysis reveals environmental and genetic determinants of element covariation in the maize grain ionome

**DOI:** 10.1002/pld3.139

**Published:** 2019-05-10

**Authors:** Alexandra Asaro Fikas, Brian P. Dilkes, Ivan Baxter

**Affiliations:** ^1^ Donald Danforth Plant Science Center St. Louis Missouri; ^2^ Washington University in St. Louis St. Louis Missouri; ^3^ Department of Biochemistry Purdue University West Lafayette Indiana

**Keywords:** genotype‐by‐environment interactions, ionomics, maize, mineral nutrients, multivariate traits

## Abstract

The integrated responses of biological systems to genetic and environmental variation result in substantial covariance in multiple phenotypes. The resultant pleiotropy, environmental effects, and genotype‐by‐environmental interactions (GxE) are foundational to our understanding of biology and genetics. Yet, the treatment of correlated characters, and the identification of the genes encoding functions that generate this covariance, has lagged. As a test case for analyzing the genetic basis underlying multiple correlated traits, we analyzed maize kernel ionomes from Intermated B73 x Mo17 (IBM) recombinant inbred populations grown in 10 environments. Plants obtain elements from the soil through genetic and biochemical pathways responsive to physiological state and environment. Most perturbations affect multiple elements which leads the *ionome*, the full complement of mineral nutrients in an organism, to vary as an integrated network rather than a set of distinct single elements. We compared quantitative trait loci (QTL) determining single‐element variation to QTL that predict variation in principal components (PCs) of multiple‐element covariance. Single‐element and multivariate approaches detected partially overlapping sets of loci. QTL influencing trait covariation were detected at loci that were not found by mapping single‐element traits. Moreover, this approach permitted testing environmental components of trait covariance, and identified multi‐element traits that were determined by both genetic and environmental factors as well as genotype‐by‐environment interactions. Growth environment had a profound effect on the elemental profiles and multi‐element phenotypes were significantly correlated with specific environmental variables.

## 1 INTRODUCTION

Elements are distinct chemical species, and studies of element accumulation frequently investigate each element separately. There is overwhelming evidence, however, that element accumulations covary due to physical, physiological, genetic, and environmental factors. In a dramatic example in *Arabidopsis thaliana*, a suite of elements responds to Fe deficiency in such a concerted manner that they can be used to predict the deficiency or sufficiency of Fe for the plant more accurately than the measured level of Fe in plant tissues (Baxter et al., 2008). The basis of this covariation can be as simple as co‐transport of multiple elements. IRT1 is a metal transporter capable of transporting Fe, Zn, and Mn. IRT1 is upregulated in low Fe conditions resulting in an environmentally dependent link between Fe and other ions (Korshunova, Eide, Clark, Guerinot, & Pakrasi, 1999). Other pairs of co‐regulated elements, such as Ca and Mg which share homeostatic pathways in *Brassica oleracea* (Broadley et al., 2008), should be affected predictably by genetic variation. When *A. thaliana* recombinant inbred line populations were grown in multiple environments, genetic correlations among Li‐Na, Mg‐Ca, and Cu‐Zn were observed across all environments while Ca‐Fe and Mg‐Fe were only correlated in a subset of environments (Buescher et al., 2010). Shared genetic regulation of ion transport without substantial environmental responsiveness should result in the former pattern, along with significantly less capacity for homeostasis across environmental concentrations and availabilities of elements. Environmentally responsive molecular mechanisms, reminiscent of *IRT1* upregulation, could result in environmentally variable patterns of correlations. Baxter, Gustin, Settles, and Hoekenga (2013) previously tested element seed concentrations for correlations in the maize Intermated B73 x Mo17 (IBM) recombinant inbred population, finding several correlated element pairs, the strongest of which was between Fe and Zn. Yet, few QTL impacting more than one element were found, possibly due to QTL with small effects on multiple elements failing to exceed the threshold of observation when mapping on single‐element traits with limited numbers of lines. Thus, while understanding the factors driving individual element accumulation is important, we must consider the ionome as a network of co‐regulated and interacting traits (Baxter, 2009). We propose that formally considering this coordination between elements can provide deeper insight than focusing on each element in isolation and that this will be a general feature of massively parallel phenotyping data and homeostatic systems.

Multivariate analysis techniques, such as principal components analysis (PCA), can reduce data dimension and summarize covariance of multiple traits as vectors of values by minimizing the variances of input factors to new components. When multiple phenotypes covary, as occurs for the elements in the ionome, this approach may complement single‐element approaches by describing trait relationships. In studies on crops such as maize, PCA has been used as a strategy to consolidate variables that may be redundant or reflective of a common state (Bouchet et al., 2017; Buescher, Moon, Runkel, Hake, & Dilkes, 2014; Burton et al., 2015; Frey, Presterl, Lecoq, Orlik, & Stich, 2016; Shakoor et al., 2016). PCA has proved useful in previous QTL mapping efforts, facilitating detection of new PC QTL not found using univariate traits in analyses of root system architecture in rice (Topp et al., 2013) and kernel attributes, leaf development, ear architecture, and enzyme activities in maize (Choe & Rocheford, 2012; Liu, Garcia, McMullen, & Flint‐Garcia, 2016; Zhang et al., 2010). In the current study, we expect that elemental variables are functionally related and therefore need new traits to describe elemental covariation. Since we do not know the exact nature of these relationships, and the ionome varies depending on environment, PCA is useful in that it does not require a priori definition of relationships between variables. If the PCA approach leads to novel loci and insights into how the ionome is functioning, it will be a valuable addition to the study of mineral nutrient regulation.

Here we show that developing multivariate traits reveals environmental and genetic effects that are not detected using single elements as traits. We performed PCA on element profiles from the maize IBM population (Lee et al., 2002) grown in 10 different environments. Different relationships between elements were identified that depended on environment. QTL mapping using multi‐element PCs as traits was carried out within each environment separately. Comparing these multivariate QTL mapping results to previous single‐element QTL analyses of the same data (Asaro et al., 2016) and demonstrates that a multivariate approach uncovers unique loci affecting multi‐element covariance. Additionally, experiment‐wide PCA performed on combined data from all environments produced components capable of separating lines by environment based on their whole‐ionome profile. These experiment‐wide factors, while representative of environmental variation, also exhibited a genetic component, as loci affecting these traits were detected through QTL mapping. This shared involvement in element covariation is the expectation of genetic and environmental variation resulting in adjustments to the physiological mechanisms underlying adaptation.

## 2 METHODS

### 2.1 Field growth and data collection

#### 2.1.1 Field growth and elemental profile analysis

Lines belonging to the Intermated B73 x Mo17 recombinant inbred (IBM) population (Lee et al., 2002) were grown in 10 different environments: Homestead, Florida in 2005 (220 lines) and 2006 (118 lines), West Lafayette, Indiana in 2009 (193 lines) and 2010 (168 lines), Clayton, North Carolina in 2006 (197 lines), Poplar Ridge, New York in 2005 (256 lines), 2006 (82 lines), and 2012 (168 lines), Columbia, Missouri in 2006 (97 lines), and Ukilima, South Africa in 2010 (87 lines). Elemental analysis was carried out in a standardized inductively coupled plasma mass spectrometry (ICP‐MS) pipeline previously described in detail (Asaro et al., 2016). Analytical outlier removal and weight normalization was performed following data collection as described in our previous analysis of these data.

### 2.2 Computational analysis

#### 2.2.1 Element correlation analysis

Within environments, 190 Pearson correlation coefficients were calculated, one for each pair of the 20 measured elements. To control for multiple tests, we applied a Bonferroni correction at an alpha level of 0.05. Given 190 possible combinations, correlations with a *p*‐value below 0.05/190 = 0.00026 were regarded as significant.

#### 2.2.2 Principal components analysis of ionome variation within environments

Elements prone to analytical error (B, Na, Al, As) were removed before to PC analysis, leaving 16 elements: Mg, P, S, K, Ca, Mn, Fe, Co, Ni, Cu, Zn, Se, Rb, Sr, Mo, and Cd. B, Na, Al, and As have a fairly low signal to noise ratio; because all elements are scaled together in a PCA, including these elements would increase the amount of noise variation going into the PCA. In an attempt to summarize the effects of genotype on covariance of ionomic components, a PCA was done using elemental data for each of the 10 environments separately. The *prcomp* function in R with scale = TRUE was used for PCA on elemental data to perform PCA on the line average element values in an environment. This function performs singular value decomposition on a scaled and centered version of the input data matrix, computing variances with the divisor *N*‐1. Sixteen PCs were returned from each environment. The IBM population is a large and diverse population and we observe extensive variation across the elements, so even a small proportion of variation could explain a substantial amount of actual variation. We used a PCA scree plot to guide our choice of a 2% cutoff (Figure S1). After removal of PCs accounting for less than 2% of the variance, the 10 sets of PCs were used as traits in QTL analysis. Variance proportions and trait loadings for all PCs calculated across 10 environments are provided in Table S3.

#### 2.2.3 QTL mapping: principal components

Quantitative trait loci mapping was done using stepwise forward–backward regression in R/qtl (Broman & Speed, 2002) as described previously for element phenotypes (Asaro et al., 2016). The mapping procedure was done for each environment separately, with PC line means for RILs in the given environment as phenotypes and RIL genotypes as input. The *stepwiseqtl* function was used to produce an additive QTL model for each PC, with the max number of QTL allowed for each trait set at 10. The 95th percentile LOD score from 1,000 *scanone* permutations was used as the penalty for addition of QTL. One thousand permutations were done for every trait–environment combination. The QTL model was optimized using *refineqtl* for maximum likelihood estimation of QTL positions. The locations of the PC QTL detected in this study were compared to the single‐element QTL from our previous study. Loci were considered distinct if they were at least 25 cm away from any single‐element QTL detected in the environment in which the PC QTL was detected. This serves as a conservative control in order to minimize the mistaken assessment of novelty for QTL with small changes in peak position.

#### 2.2.4 QTL by environment analysis: PCA across environments

The 16 most precisely measured elements were used for an additional principal components analysis. Again, the *prcomp* function in R with scale = TRUE was used for PCA on elemental data; however, all 16 element measurement values in all lines in all of the 10 environments were combined into one PCA. These PCs are referred to as across‐environment PCs (aPCs). Element loadings were recorded and plotted along with lines colored by environment for aPCs 1 and 2 (Figure S4). The first seven aPCs explained 93% of the total covariation of these traits. A linear model was used to test the relationship of environmental parameters on these aPCs. All seven aPCs were also used for stepwise QTL mapping by the same method described above, with 1,000 permutations for every trait–environment combination used to set 95th percentile significance thresholds.

#### 2.2.5 QTL by environment analysis: projection‐PCA across environments

The sets of lines grown in each our 10 environments were drawn from the same population (Lee et al., 2002) but different subsets were grown and harvested in different environments. To achieve common multivariate summaries for all lines and growouts, we performed an alternative PCA using a smaller set of common lines. We then projected the loadings from this PCA onto the full dataset, as follows. First, a PCA was conducted on 16 lines common to six of the 10 environments (FL05, FL06, IN09, IN10, NY05, NY12). The loadings for each PC from this PCA were then used to calculate values from full set of lines across 10 environments to generate PCA projections (PJs). These derived values based on a common‐line PCA were compared to previously described aPC values from the PCA done on all lines at once. Correlations between PJs and aPCs were computed to compare the outcomes of the two methods.

#### 2.2.6 Weather and soil data collection and analysis

Weather data for FL05, FL06, IN09, IN10, NC06, NY05, NY06, and NY12 were downloaded from Climate Data Online (CDO), an archive provided by the National Climatic Data Center (NCDC) through the National Oceanic and Atmospheric Administration (http://www.ncdc.noaa.gov/cdo-web/). Data were not available for the South Africa growout. Daily summary data for each day of the growing season were tabulated from the weather station nearest to the field location. Weather stations used to obtain data for each location are indicated in Table S4. Minimum temperature (in degrees Celsius) and maximum temperature (in degrees Celsius) were available in each location. With these variables, average minimum temperature, and maximum temperature were calculated across the 120‐day growing season as well as for 30 day quarters. Growing degree days (GDD) were calculated for the entire season and quarterly using the formula GDD = ((*T*_max_ + *T*_min_)/2) − 10. No max or min thresholds were used in the GDD calculation.

Data describing soils from each location were obtained from the Web Soil Survey provided by the USDA Natural Resources Conservation Service (http://websoilsurvey.sc.egov.usda.gov/App/HomePage.htm). A representative area of interest was selected at the site of plant growth using longitude and latitude coordinates. When an area contained more than one soil type, a weighted average of measurements from all soil types was used. The data we downloaded from the Web Soil Survey were: pH, electrical conductivity (EC) (decisiemens per meter at 25 degrees C), available water capacity (AWC) (centimeters of water per centimeter of soil), available water supply (AWS) (centimeters), and calcium carbonate (CaCO3) content (percent of carbonates, by weight). Layer options were set to compute a weighted average of all soil layers.

The relationships between the seven experiment wide aPCs and the weather and soil variables were estimated by calculating Pearson correlation coefficients for the pairwise relationships. Correlations were also calculated between average element values and soil and weather variables in each environment.

## 3 RESULTS

### 3.1 Summary of data collection and previous analysis of single‐element traits

We previously acquired data on 20 elements measured in the seeds from *Zea mays* L. Intermated B73 x Mo17 recombinant inbred line (IBM) populations (Lee et al., 2002) grown in 10 different location/year settings (Asaro et al., 2016). This work is briefly summarized here as it serves as the basis of our comparison. The kernels came from RILs of the IBM population cultivated across six locations and 5 years. Quantification of the accumulation of 20 elements in kernels was done using inductively coupled plasma mass spectrometry (ICP‐MS). Weight‐adjusted element measurements were used for a QTL analysis to detect loci contributing to variation in seed element contents (Asaro et al., 2016). The current study is motivated by previous demonstrations of elemental correlations and mutant phenotype analyses which indicate extensive relationships between elements (Baxter et al., 2008; Buescher et al., 2010). To explore this formally, we further analyzed these data from a multiple‐element perspective.

### 3.2 Element to element correlations

**Figure 1:**
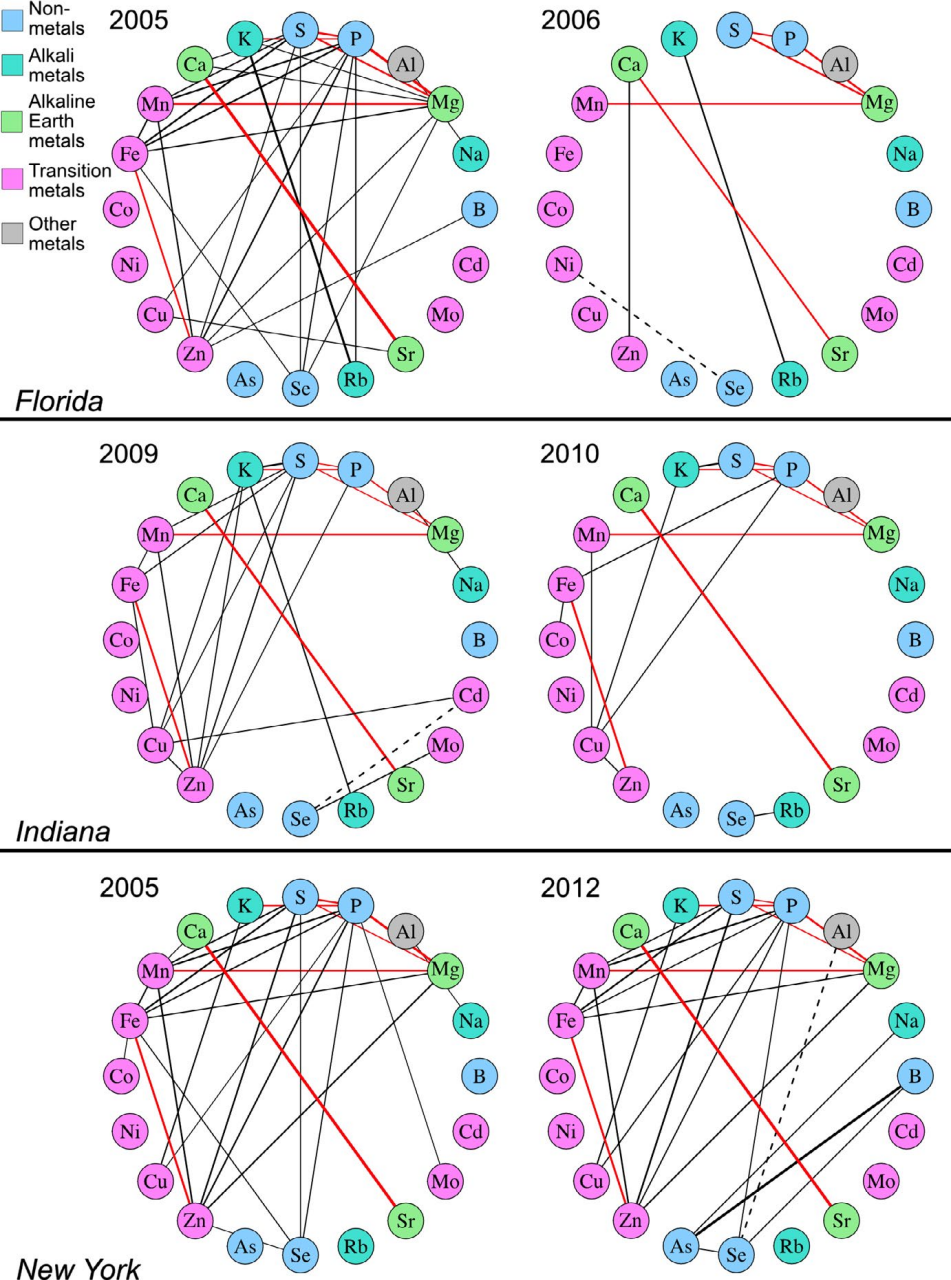
Element correlations diagrams for locations with repeated measurements. Pairwise correlations of 20 kernel elements in varying environments, shown for the experiments within locations having data from multiple years (FL, IN, and NY). Correlations were calculated as the Pearson correlation coefficient (*r*_p_) between concentration values for each element pair. Significance was evaluated using a Bonferroni correction for multiple tests within each environment and set at a corrected *p* value of 0.05. Lines between elements represent significant pairwise correlations, weighted by strength of correlation. Positive and negative correlations are represented as solid and dashed lines, respectively. Red lines indicate correlations present in at least 5 of the 6 environments shown

Several elements were highly correlated across the dataset, exhibiting pairwise relationships among lines in a given environment that passed a conservative Bonferroni correction for multiple tests. Many of these correlations reflected results previously obtained by Baxter et al. (2013), such as the strong correlation between Fe and Zn. We detected 209 pairs of elements that were genetically correlated out of 1,900 possible correlations across environments (190 pairs per environment). We expect robust genetic influence to produce repeated observation of trait correlations in multiple environments. Of the six locations included in this experiment, we obtained data from three locations (FL, IN, and NY) from plant material grown in two different years. Seven element‐pairs were correlated in five or more of these six environments: Mn and Mg, Mg and S, Mg and P, S and P, P and K, Ca and Sr, and Fe and Zn (Figure 1). Other element‐pair correlations were driven by the genetic variation between IBM RIL in fewer environments. For example, Mn and P were correlated in FL05, NY05, and NY12 (*r*_p_ = 0.50, 0.48, 0.51) but were not significantly correlated in FL06, IN09, or IN10 (*r*_p_ = 0.31, 0.20, 0.18). Thus, while some correlations exist in multiple years and multiple locations, element correlations were affected by both location and year.

**Figure 2:**
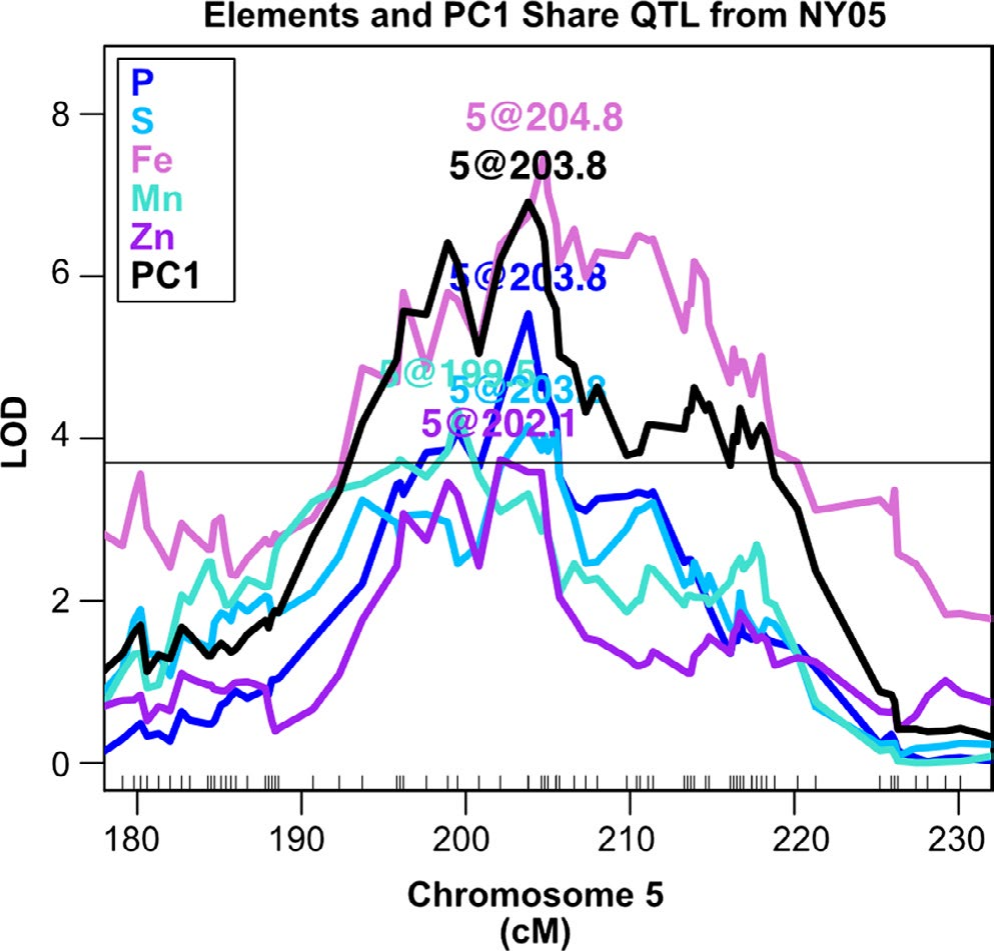
Multiple‐element QTL. Stepwise QTL mapping output from the NY05 population for P, S, Fe, Mn, Zn, and PC1. Position in cM on chromosome 5 is plotted on the *x*‐axis and LOD score is shown on the *y*‐axis. Ninety‐fifth percentile of highest LOD score from 1,000 random permutations is indicated as horizontal line

In our previous single‐element QTL analysis of these data, loci comprising QTL for two or more different elements were detected (Table 1). This mutual genetic regulation of multiple elements was readily apparent in the trait correlations calculated within environments, as five of the nine shared‐element QTL exhibited corresponding element‐pair correlations within the given environment. For example, phosphorous, which was in three of the seven most reproducible element‐pair correlations, exhibited the highest incidence of shared QTL with other elements. These included common QTL between P accumulation and all three of the reproducibly P‐correlated elements: S and the cations K and Mg. In addition, P was affected by the only QTL shared between more than two elements, which affected P, S, Fe, Mn, and Zn accumulation in NY05 (Figure 2). Consistent with the possibility of variation in transport processes affecting element accumulation correlations, overlapping QTL were frequently found between elements with similar structure, charge, and/or type, such as Ca and Sr or Fe and Zn. These element correlations and post hoc comparisons of shared QTL localizations suggest a genetic basis for covariance of the ionome in the RIL population.

**Table 1:** Loci affecting variation for multiple elements in the same environment

Environment	Chr	Pos (cM)^a^	El 1	El 2	El 3	El 4	El 5
NY05	1	400	Mn	Ni	—	—	—
NY05	3	323	Sr	Ca	—	—	—
NY05	5	201	Mn	Zn	P	S	Fe
NY06	1	532	Mn	Mg	—	—	—
IN09	4	306	Fe	K	—	—	—
IN10	2	213	Mo	Cd	—	—	—
NY12	5	203	Zn	Fe	—	—	—
FL05	1	230	B	Mn	—	—	—
FL05	4	159	Fe	Zn	—	—	—

a Average position.

### 3.3 Principal components analysis of covariance for elements in the ionome

To better describe multi‐element correlations and thereby detect loci controlling accumulation of two or more elements, we derived summary values representing the covariation of several elements. We implemented an undirected multivariate technique, principal components analysis (PCA), for this purpose. PCA reduced co‐varying elements into principal components (PCs), orthogonal variables that account for variation in the original dataset, each having an associated set of rotations (also known as loadings) from the input variables. After removing elements prone to analytical artifacts, PCA was conducted using the remaining 16 elements from each of the 10 environments separately. This produced 16 principal components in each environment (Figure S1) of which we retained for further analysis only PCs representing more than 2% of the total variation. This resulted in as few as 11 and as many as 15 PCs depending on environment.

**Figure 3:**
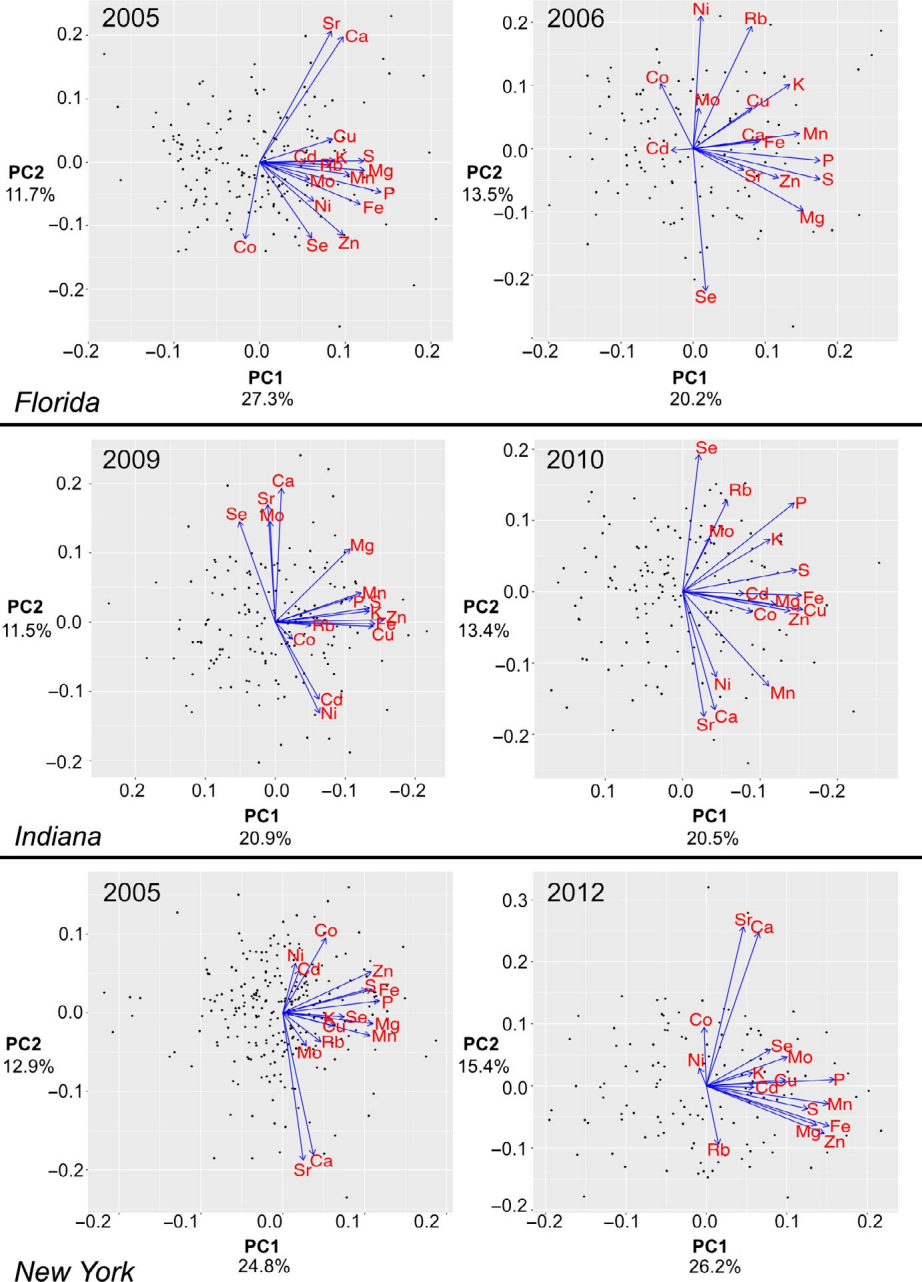
PCA plots in multiple environments. PCA plots showing PC1 and PC2 loadings in different years in three locations (FL, IN, and NY). PC1 and PC2 values for each line are plotted as points and PC1 and PC2 loadings of each element are indicated by blue arrows. The data for different years for each of three locations, FL, IN, and NY are plotted. The percent of total variation explained by each PC is labeled on the axes. PC negative and positive values are arbitrary, so the Indiana *x*‐axes are switched in direction to aid visual comparisons

Remarkably, there is substantial overlap in the loadings of many elements in the first and second PCs across some environments, suggesting a reproducible effect of genetic variation on the covariance of elemental accumulation in these environments (Figure 3). Additionally, the loadings of elements are consistent with the pair wise relationships observed in the element‐by‐element correlations. For example, the chemical analogs Ca and Sr frequently load PCs in a similar direction. The PC loadings derive from inputs of several elements to a single PC variable. All retained PCs in all 10 environments have a loading contribution of at least 0.25 in magnitude from two or more elements. While some patterns existed across environments, many PC loadings differed in both magnitude and direction according to environment. This suggests instability of element‐pair correlations across the environments. We used correlation tests of element loadings to detect PCs stemming from shared biological processes in each environment. This identified PCs from each environment were constructed from similar relationships. Because loading direction is arbitrary, both strong positive and strong negative correlations were examined. Fifty‐two pairs of PCs exhibited loadings correlations with a Pearson correlation coefficient greater than 0.75 or less than −0.75 (Figure S2). Thus, the PC analyses of data across different locations described similar patterns of elemental covariation, while not necessarily recovered in the same rank order.

### 3.4 QTL mapping of ionomic covariance components

**Figure 4:**
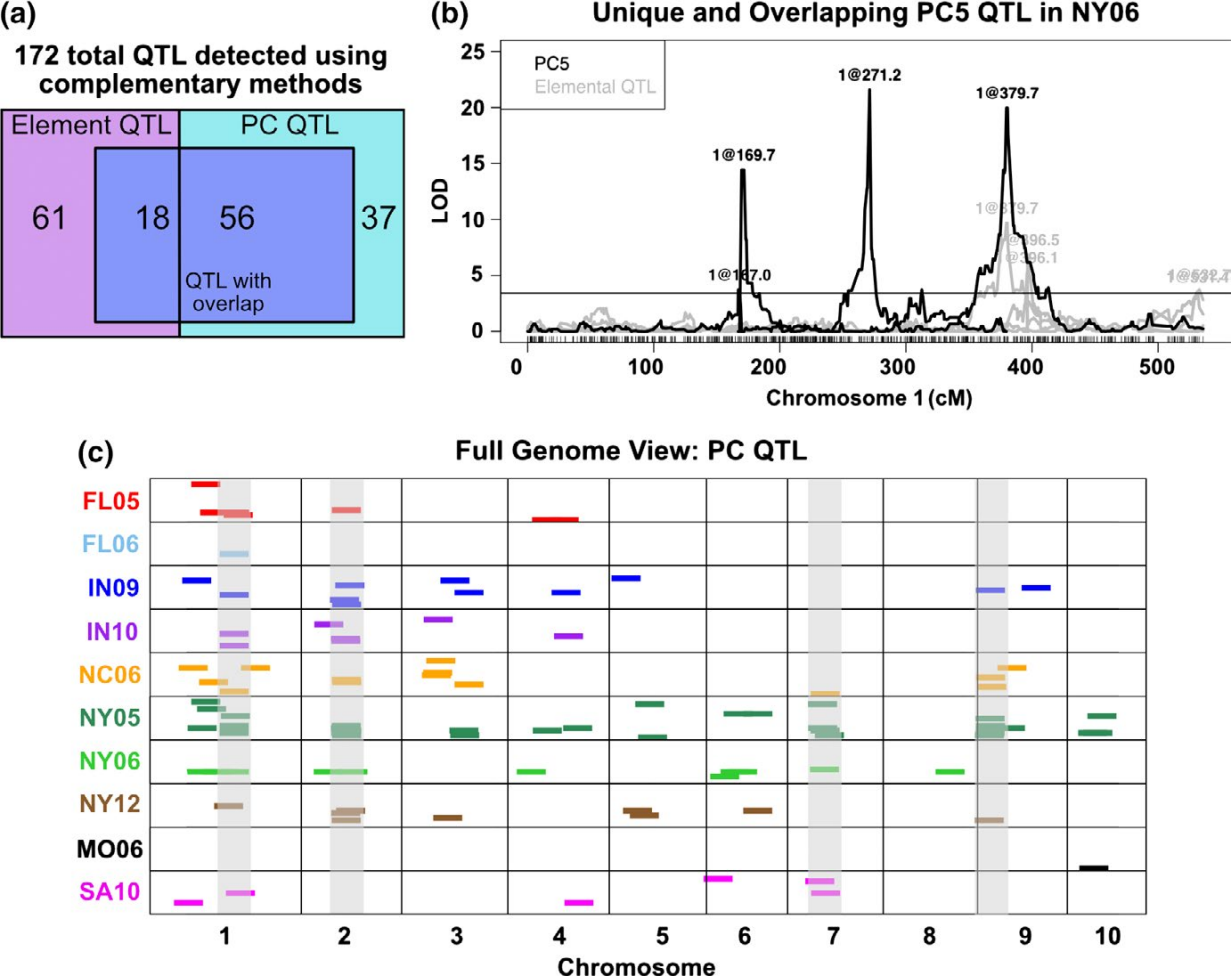
Principal component QTL from 10 environments. PCs were derived from elemental data separately in each of 10 environments and used as traits for QTL mapping. (a) One hundred and seventy‐two total element and PC QTL were mapped. The two boxes represent the 79 and 93 elemental and PC QTL, respectively. Eighteen element QTL overlap with PC QTL from the same environment. Fifty‐six PC QTL overlap with element QTL from the same environment. Sets of non‐unique QTL are shown in the center box. QTL unique to elements, 61, and to PCs, 37, are shown outside of the shared box. (b) QTL mapping output for PC5 from the NY06 population. Position on chromosome 1 is shown on the *x*‐axis, LOD score is on the *y*‐axis. All significant NY06 element QTL on chromosome 1 are shown in grey (α = 0.05). Two PC5 QTL, at 169.7 and 271.2 cM, are unique to PC5 and do not overlap with any elemental QTL. A PC5 QTL at 379.7 cM is shared with a molybdenum QTL. (c) Significant PC QTL (α = 0.05) for PCs in 10 environments. QTL location is shown across the 10 chromosomes on the *x*‐axis. Environment in which QTL was found is designated by color. QTL are represented as dashes of uniform size for visibility. Four regions highlighted in grey represent the four loci found for multiple PC traits in multiple environments (>2)

The PCs from each environment were used as traits for QTL detection. Stepwise QTL mapping using these derived traits yielded 93 QTL that exceeded an statistical threshold of *α* = 0.05 estimated by 1,000 permutations performed for each trait–environment combination (Figure 4c). QTL were found for PC traits explaining both large and smaller proportions of variation (Table S1). Fifty‐six of these QTL affecting multiple‐element covariance components overlapped with previously detected single‐element QTL in the same environment (Asaro et al., 2016) (Figure 4a). In some cases, two or more PC traits within an environment resolved to one single‐element QTL. This was observed particularly for elements with strong effect QTL, such as Mo, Cd, and Ni. For example, in IN10, PC2, and PC10 both have QTL that co‐localize with the Cd QTL on chromosome 2. Likewise, in NY05, PC3, PC5, PC6, and PC9 all detect QTL coinciding with the large‐effect Ni QTL on chromosome 9. Each of these PCs are comprised of varying loadings of Ni, along with other elements. This demonstrates that, although the relationship among elements described by each PC is distinct, a locus affecting a single‐element can be detected due to loading of that element into more than one PC. This repeated detection of the same locations contributes to the higher number and proportion of detected PC QTL that were shared with element QTL (56/93) than element QTL that were shared with PC QTL (18/79), although the same genomic locations underlie this overlap.

Quantitative trait loci mapping on single elements may not have the power to detect loci with small coordinate effects on several elements. PC traits can reveal new QTL and enhance detection of common genetic factors modulating elements. Thirty seven PC QTL were detected at loci not seen using single‐element traits. For instance, two PC5 QTL from the NY06 growout were located on chromosome 1 at positions distinct from any single‐element QTL (Figure 4b). So as to not inflate PC‐specific QTL, they are defined here as QTL greater than 25 cm away from any elemental QTL in the same environment. Top elemental loadings of PCs and the overlap with elemental QTL is summarized in Table S2.

Principal component QTL analysis captured previously observed single‐element QTL shared between elements within a particular environment. Of the nine loci affecting variation for multiple elements in the same environment (Table 1), four loci were detected for a PC trait in that environment (Table 2). For example, in NY05, a QTL was identified for PC1 that overlaps QTL that were detected in the single‐element analyses of P, S, Fe, Mn, and Zn on chromosome 5 (Figure 2). The log of odds score for this NY05 PC1 QTL was as strong as the association between the locus and Fe accumulation and more significant than the P, S, Mn, and Zn elemental QTL. Thus, the QTL for a multi‐element PC was as strong as the best single‐element approach for this previously detected multi‐element locus. This is the prediction for traits that will affect variation in multiple elements, such as root structure or homeostatic processes. For these traits, the PC approach may be preferable to single elements, particularly in cases where single‐element changes are of small effect or below detection limits while concerted changes to multiple elements display a larger effect.

**Table 2:** Loci associated with multiple elements and PC(s) in the same environment

Environment	Chr	Pos (cM)^a^	Elements	PC(s)
NY05	1	400	Mn, Ni	PC11
NY05	3	323	Sr, Ca	—
NY05	5	201	Mn, Zn, P, S, Fe	PC1
NY06	1	532	Mn, Mg	—
IN09	4	306	Fe, K	—
IN10	2	213	Mo, Cd	PC2, PC4
NY12	5	203	Zn, Fe	PC7
FL05	1	230	B, Mn	—
FL05	4	159	Fe, Zn	—

a Average position of all element QTL, PC QTL are within 5 cM.

Comparing PCs from different environments identified 52 PC pairs with similar loadings. Of these, 37 had no QTL for one or both of the PCs, consistent with a common environmental factor variable in those fields as the basis of that variation. Of the remaining 15 pairs, for which at least one QTL was detected for each member of the pair, five pairs had QTL that co‐localized. In all five cases, the QTL overlapping between these pairs of PCs correspond to a large‐effect single‐element QTL. Six PC traits belonging to three correlated pairs, PC4 in NY05 and PC6 in IN09 (*r*_p_ = 0.81), PC4 in FL05 and PC3 in NY05 (*r*_p_ = −0.84), and PC3 in IN10 and PC2 in NC06 (*r*_p_ = 0.89), detected a QTL coinciding with a Mo QTL, a locus on chromosome 1 encoding the ortholog of the *A. thaliana* MOT1 molybdenum transporter. The same scenario exists for PC2 in IN09 and PC2 in NY05 (*r*_p_ = −0.78), both affected by the QTL on chromosome 2 that had a strong effect on Cd in our single‐element QTL mapping experiments. Finally, PC8 in NC06 and PC5 in NY05 (*r*_p_ = 0.76) both map to a large‐effect Ni QTL. Despite the resolution to QTL detected in a single‐element analysis, in all of these cases, correlations between loadings were not driven by a single element, but rather by similar loadings for most elements (Figure S2). In addition to overlaps at these strong‐effect single‐element QTL, six other pairs of correlated PCs have QTL that do not overlap. Correlated PCs with QTL at different chromosomal positions in different environments could be due to states, such as iron deficiency, that may arise from distinct processes in each environment (e.g., soil pH or low Fe content) yet will generate a consistent physiological response. In these cases, the ionome displays similar trait covariance but different genetic architecture consistent with genotype by environment interactions.

**Figure 5:**
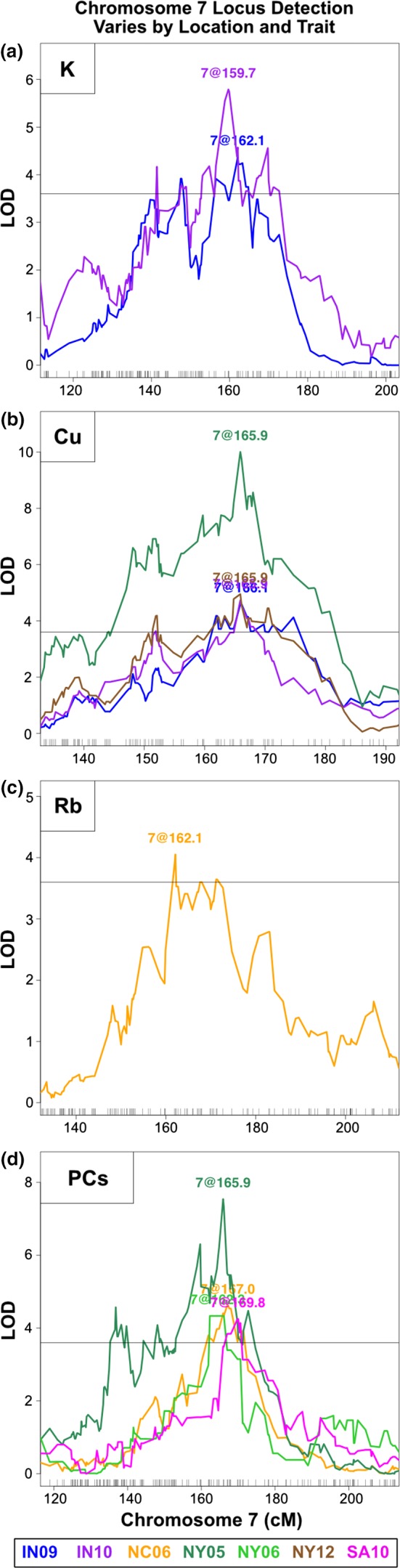
Chromosome 7 locus detection varies by location and trait. LOD score traces for QTL detected within a window on chromosome 7. Traces are colored by environment. (a) QTL for K in IN09 and IN10. (b) QTL for Cu in IN09, IN10, NY05, and NY12. (c) QTL for Rb in NC06. (d) QTL for PCs in NC06, NY05, NY06, and SA10

The PC approach also detected a QTL that was found for different single elements depending on environment. The same region on chromosome 7 was identified as a QTL for three different elements in varying environments: K in IN09 and IN10 (Figure 5a), Cu in IN09, IN10, NY05, and NY12 (Figure 5b), and Rb in NC06 (Figure 5c). In the mapping of QTL affecting the PC traits, we detected QTL at this position in some of the same environments as the single‐element QTL, NC06, and NY05, as well as in new environments, NY06 and SA10 (Figure 5d). In SA10, no QTL were mapped for Cu, Rb, or K alone. Yet, this locus was detected as significantly affecting variation in PC9 calculated from SA10, the loadings of which show a strong contribution from Cu and Rb. Likewise, in NY06, no QTL were mapped for Cu, Rb, or K, however, this locus was detected using PC6 in NY06 which has a strong loading contribution from K. No PC QTL were detected at the locus in NY12, IN09, or IN10. Thus, using PC traits in addition to single‐element traits can provide an improved estimate across different environments for the genetic effect on phenotypic variance for multi‐element loci.

The identification of both unique and previously observed QTL through this multivariate approach demonstrates the complementary nature of working with trait covariance as well as the component traits and supports previous work showing that elemental traits are mechanistically interrelated. The repeated finding of results consistent with GxE led us to investigate this formally.

### 3.5 QTL by environment interactions

Our prior analyses found QTL by environment interactions contributing to accumulation of single elements (Asaro et al., 2016). Given element correlations and partially overlapping sets of element and PC QTL, we expect to detect QTL by environment interactions that impact multi‐element traits. To look at the effects of environment on genetic regulation of multi‐element phenotypes, we conducted another PCA, this time on element concentrations of lines from all environments combined. If the genetic and environmental variances do not interact, we expect some PCs will reflect environmental variance and others will reflect genetic variance. However, if the ionome is reporting on a summation of physiological status that results from genetic and environmental influences, some PCs calculated from ionomic traits should be both correlated with environmental factors and result in detectable QTL.

#### 3.5.1 PCA across environments

The covariance between element accumulation data across all environments was summarized using principal components analysis. Elements prone to analytical artifacts (B, Na, Al, As) were removed prior to analysis. Sixteen across‐environment PCs (aPCs) describing the covariation of the ionome were calculated for every RIL in every environment.

Out of a concern that the different lines present in each growout unduly influenced the construction of PCs specific to each environment, we performed the following tests. First, we looked at only those locations where two or more growouts were performed, so that location replication might be considered. Second, to identify a balanced sample set present in all environments, we identified the lines that were grown in all of these six growouts. PCA of the 16 element measurements was conducted across environments (Figure S3) and the loadings of each element into each PC were recorded. Thus, the loadings of the 16 elements in the PCA were calculated from a set of common genotypic checks distributed within each environment. We used these loadings to calculate PCA projections (PJs) from all lines in all environments. In this way we made comparisons of the same calculated values in each environment. We found that the PJs and aPCs were strongly correlated; PJ1 and aPC1 were nearly identical (*r*_p_ = 0.998) and PJs 2–5 correlated with at least one of aPCs 2–5 at *r*_p_ > 0.66. The correlations between the loadings from PJs and aPCs reflected these same patterns. To reduce the incidence of artifacts or overfitting, aPCs accounting for less than 2% of the total variation were eliminated for further analyses, leaving seven aPCs.

**Figure 6:**
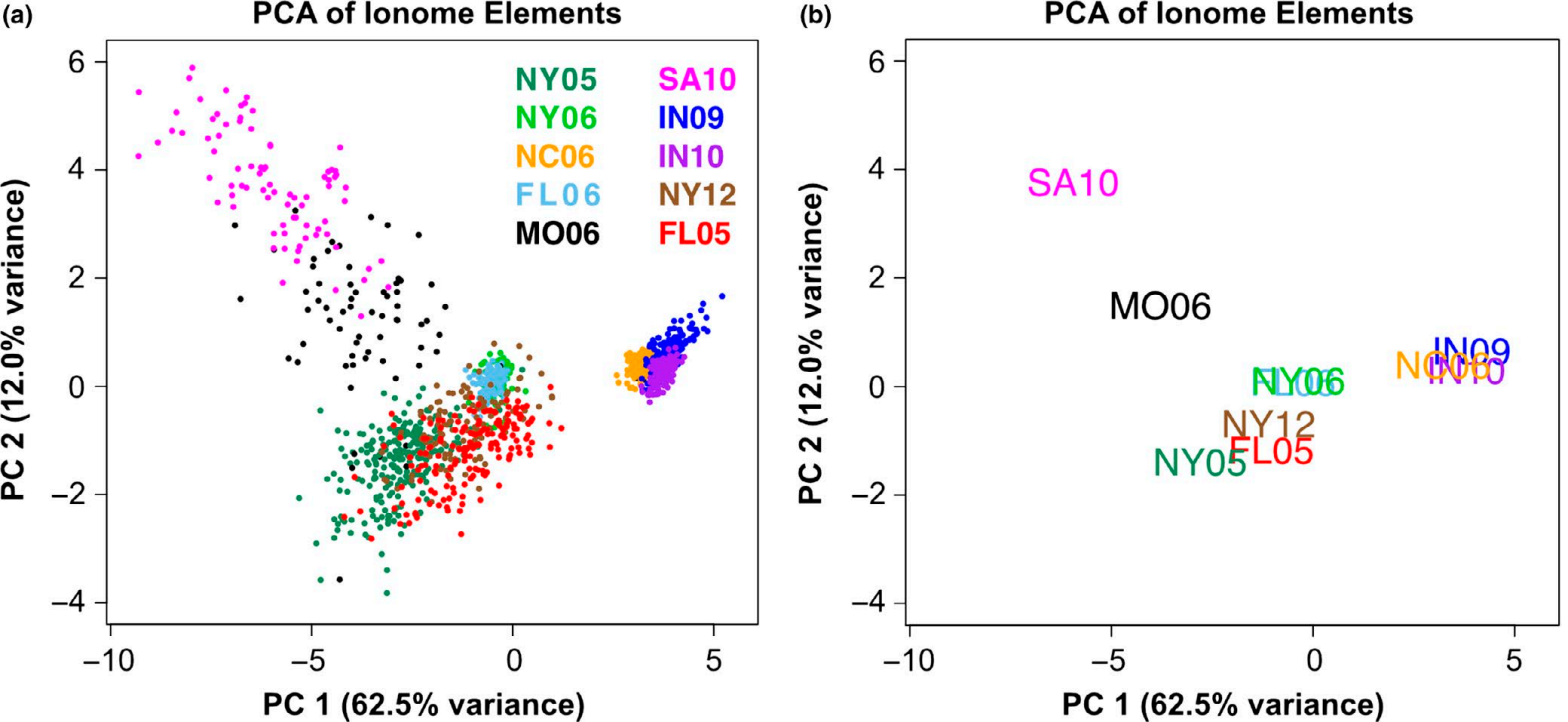
PCA Separates Lines by Environment. PC1 and PC2 separate lines by environment. Points correspond to lines, colored by their environment. (a) Across‐environment PC1 versus PC2 values for each line, colored by environment. Percentage of total variance accounted for by each PC indicated on the axes. (b) Average across‐environment PC1 versus PC2 values for all lines in each environment

**Figure 7:**
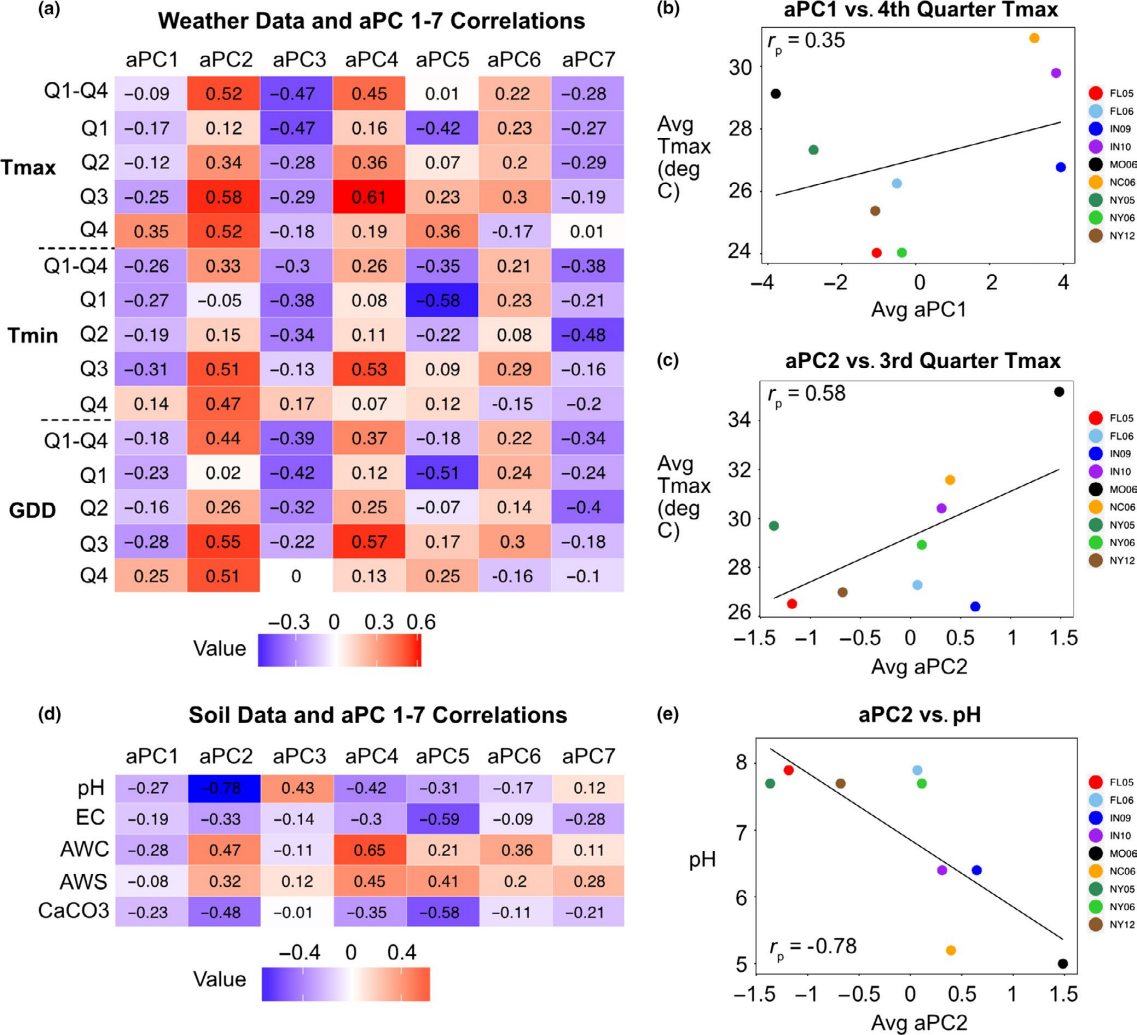
aPC and Weather Variable Correlations. (a) Heatmap showing Pearson correlation coefficients (*r*_p)_ between averaged aPC 1–7 values across environments and averages for maximum temperature, minimum temperature, and GDD across the growth season and for each quarter of the season. Red and blue intensities indicate strength of positive and negative correlations, respectively. (b) Average aPC1 values for nine environments versus average maximum temperature for each environment over the fourth quarter of the growing season. Points colored by environment. Pearson correlation coefficient is shown within the graph. (c) Average aPC2 values for nine environments versus average maximum temperature for each environment over the third quarter of the growing season. (d) Heatmap showing correlations between aPCs 1–7 and soil attributes: pH, electrical conductivity (EC), available water capacity (AWC), available water storage (AWS), and calcium carbonate (CaCO3). (e) Average aPC2 values versus pH

Growth environment had a significant effect on all aPCs (*p* < 0.001). The first two aPCs were highly responsive to the environment (Figure 6). The lines from each environment cluster together when plotting aPC1 vs aPC2 values, with distinct separation between environments and years. In order to identify environmental factors responsible for ionome covariance, weather station and soil data from all environments except SA06 were recovered from databases (see Section 2). Correlations were calculated between season‐long or quarter‐length summaries of temperature and the aPC values for the nine environments. The weather variables, all temperature‐based, were not correlated with aPCs in many cases, although correlations exceeding *r*_p_ = 0.50 were observed for aPCs 2,4, and 5 (Figure 7a). The strongest correlation observed for aPC1 was with average maximum temperature in the fourth quarter of the growing season (*r*_p_ = 0.35) (Figure 7b) while the highest observed for aPC2 was for average maximum temperature during the third quarter (*r*_p_ = 0.58) (Figure 7c). The relatively small number of environments, substantial non‐independence of the weather variables, and likely contribution of factors other than temperature limit the descriptive power of these correlations.

The lack of particularly strong correlations between the first two aPCs and temperature variables suggests that other variables, possibly field‐to‐field variation in soil composition, fertilizer application, humidity, or abiotic factors, are likely to have an influence. Correlations were also calculated between environment averages of the PCs and soil variables (Figure 7d). While the majority of these features were not found to be highly correlated with aPCs, we did observe a strong negative correlation between aPC2 and soil pH (*r*_p_ = −0.78) (Figure 7e).

**Figure 8:**
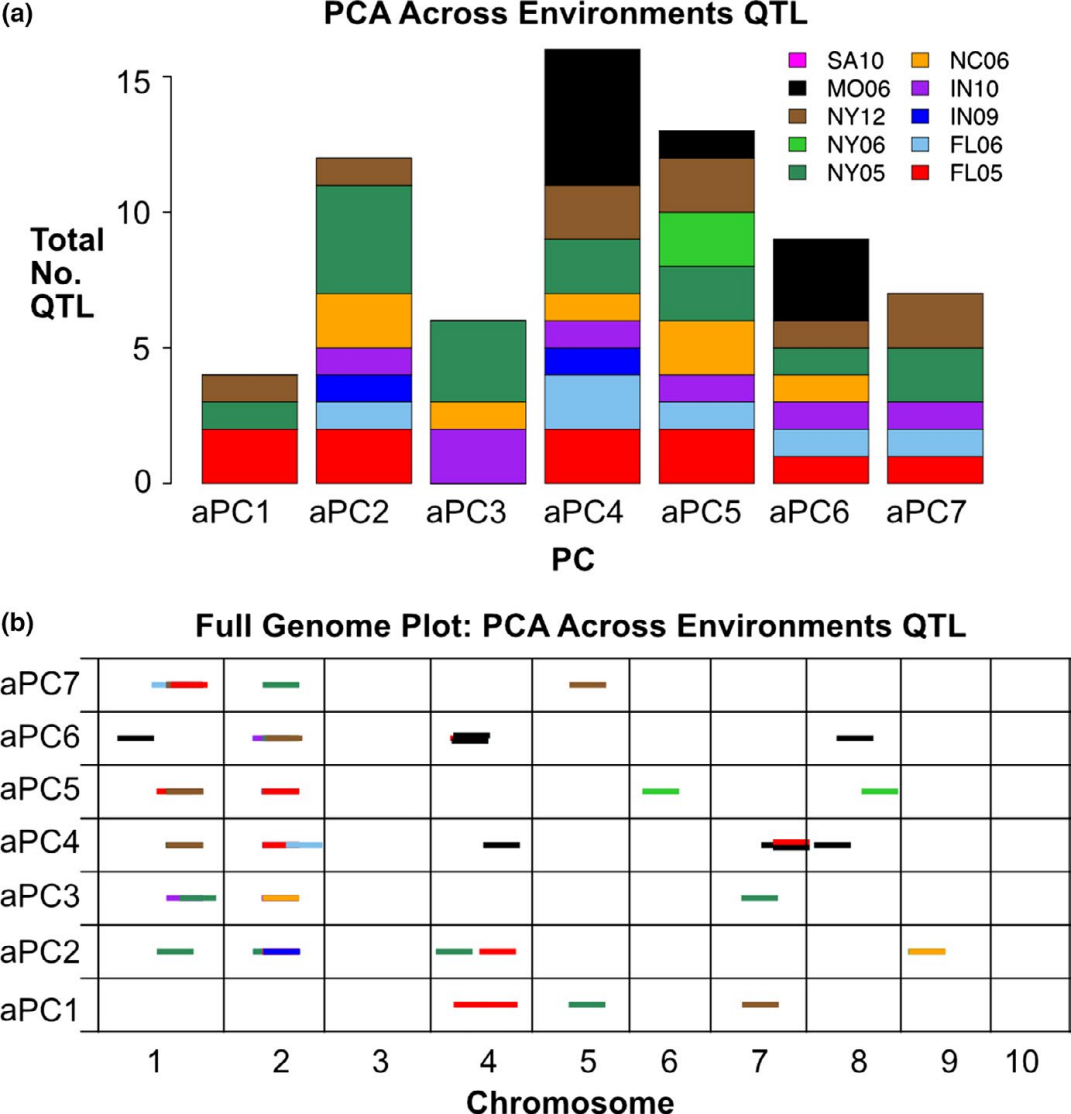
Across‐environment PCA QTL in 10 environments. QTL identified for across‐environment PCA traits (aPCs 1–7). (a) Total number of QTL detected for each aPC, colored by environment. (b) Significant QTL (*α* = 0.05) for aPCs 1–7. QTL location is shown across 10 chromosomes (in cM) on the *x*‐axis. Dashes indicate QTL, with environment in which QTL was found designated by color. All dashes are the same length for visibility

In order to determine genetic effects on these components, the calculated values for aPC1 through aPC7 were used as traits for QTL analysis in each of the 10 environments. Unlike the earlier described PCAs done in environments separately, these aPCs are calculated across all environments and are therefore comparable between environments. QTL mapping detected at least four loci controlling each aPC and a total of 38 QTL. Nine of these QTL were found in common across multiple environments and 29 were only detected in a single environment (Figure 8). Of the aPC QTL, the highest LOD score QTL were present in multiple environments and corresponded to the locations of the two strongest single‐element QTL previously detected from the same data (Mo on chromosome 1 and Cd on chromosome 2). The detection of QTL, together with the strong environmental determination of aPCs 1–7, demonstrates that ionomic covariation results from coordinate environmental and genetic variation.

Based on the stochastic detection of QTL in only a subset of growth environments, substantial interaction between the environment aPC QTL is expected. A QTL of particular interest is the aPC2 QTL detected for Mo at the ortholog of the *MOT1* locus. Previous studies have demonstrated a connection between pH and molybdenum, with Mo availability in soil being increased by high pH. It was found that the *MOT1* locus in *A. thaliana* determines response to pH changes and resultant changes in Mo availability in an allele‐specific manner, suggesting an adaptive role for variation in *MOT1* with respect to soil pH (Poormohammad Kiani et al., 2012). The correlation between aPC2 and pH was significant and aPC2 identified a QTL coinciding with a Mo QTL suggesting genetic variation in pH‐dependent changes to Mo availability across environments. The loading magnitude for Mo into aPC2 is 0.21 but Co, Ni, Rb, and Cd contribute even more, with loading magnitudes of 0.24, 0.46, 0.55, and 0.41, respectively. QTL for aPC2 also overlap with QTL for Cd and Ni. With aPC2 representing several elements, the correlation with soil pH and overlap with single‐element QTL may reflect a multi‐element phenotype responding to changes in pH. Further investigation is needed to molecularly identify the genes underlying aPC QTL, their biological roles, and their interaction with specific environmental variables.

## 4 DISCUSSION

In this study, we demonstrate that multi‐trait analysis is a valuable approach for understanding the ionome. The ionome is a homeostatic system, and effects on one element can affect other elements (Baxter et al., 2008). Many biological processes in maize have the potential to impact several elements. Indirect effects on a suite of elements have been demonstrated for numerous physiological states. Radial transport of nutrients is influenced in part by endodermal suberin, the structure and deposition of which can adapt in a highly plastic manner in response to deficiencies in K, S, Na, Fe, Zn, and Mn, potentially modifying transport of additional elements (Barberon et al., 2016). Other examples of indirect effects can be found in Arabidopsis *TSC10A* mutants with reduced 3‐ketodihydrosphinganine (3‐KDS) reductase activity. Because 3‐KDS reductase is needed for synthesis of the sphingolipids that regulate ion transport through root membranes, these mutants exhibit a completely root‐dependent leaf ionome phenotype of increased Na, K, and Rb, and decreased Mg, Ca, Fe, and Mo (Chao et al., 2011).

In line with the abundance of concerted element changes seen in ionome mutants, we detected elemental correlations and QTL that were present for more than one element. Covariance observed in elements with similar orbital configurations, such as Ca and Sr or K and Rb, is expected due to related bonding properties and functions in redox reactions. The alkali metals K and Rb have been shown to display nearly identical absorption and distribution patterns (Lauchli & Epstein, 1970). Other elements are linked through co‐regulation or common biological pathways. Phosphorous is a central nutrient in plant development and regulates other elements, complexing with cations in the form of phytic acid in maize seeds (Lopez‐Arredondo, Leyva‐González, González‐Morales, López‐Bucio, & Herrera‐Estrella, 2014). Phosphorous exhibited the greatest number of QTL overlap with other elements, including the cations K and Mg. Additional co‐localized QTL included those between Zn and Fe, Mo, and Mn, and the chemical analogs Ca and Sr. Zn and Fe can bind to the same metal transporters and metal‐binding proteins and are thus reciprocally influenced in states of excess or deficiency (Baxter, 2009; Lin et al., 2009). Three out of three of the Zn QTL that overlap with other elements involved overlap with Fe, demonstrating the genetic covariance of these elements. Mo and Mn have common roles in protein assimilation and nitrogen fixation (Mulder, 1948; Mulder & Gerretsen, 1952) and exhibit a regulatory relationship (Millikan, 1948) which may explain their overlapping genetic features. The shared QTL detected in this study likely reflect genetic polymorphisms affecting the activity of multi‐element regulatory genes or genetic changes targeted to a single element with pleiotropic effects on other elements due to homeostatic mechanisms or through concurrent multi‐element behavior.

The 37 PC‐specific loci identify loci in maize with the potential to expand our understanding of the genetic basis of ionome variation. The small population sizes used here limit our ability to interpret QTL‐effect sizes, as overestimation of QTL effect, i.e., Beavis effect, is expected. Still, the large‐effect QTL detected in our previous analysis (Asaro et al., 2016) reappear as PC QTL. There is no reason to think that effect‐size estimation will be any more accurate for PC than for single elements but careful simulations of correlated traits would be needed to demonstrate this. Regardless, it seems likely that the loading of elements into PC will make these traits just as subject to effect size overestimation and may not provide additional support for the large size.

However, in the previous single‐element QTL analysis with this same dataset, we tested for overestimation using a more stringent permutation threshold and retained 31 of 63 location‐specific QTL using a 99th percentile threshold. Biological mechanisms involving multi‐element processes or synchronized element adjustments may drive the detection of unique PC QTL. For example, the ionome has been shown to exhibit tissue‐dependent, multi‐element changes in response to nitrogen availability (Chu et al., 2016). A unique PC QTL could be detected at a nitrogen metabolism gene if variation at that gene confers additive effects on multiple elements. Variation in genes involved in adaptive responses to drought stress, soil nutrient deficiencies, or toxic micronutrient levels, can result in covariation among several elements without particularly strong effects on a single element (Baxter, 2009; Baxter & Dilkes, 2012; Baxter et al., 2008), making such genes only identifiable as QTL when working with multivariate traits.

The majority of molecularly identified ionomic mutants have multi‐element effects. In particular, mutants in genes involved in Casparian strip function and associated root‐based element flow, including *MYB36* (Kamiya et al., 2015), *ESB1* (Baxter et al., 2009), and *LOTR1* (Li et al., 2017), all display pleiotropic effects on multiple element accumulation in the leaves. In some cases, QTL affecting these traits might be detected using both single‐ and multi‐element approaches, as was the case with the chromosome 5 QTL we mapped for P, S, Fe, Mn, and Zn, as well as for PC1. However, if the changes to a suite of elements are small for individual elements or uncontrolled environmental conditions inflate the magnitude of error in measuring the genetic effects, a multi‐ionomic trait may be a better fit for QTL detection. The fact that we detect both overlapping and unique sets of element and PC QTL suggests that single and multivariate approaches should be used in concert to avoid gaps in our understanding of element regulatory networks. The evidence suggests that some of the most interesting ionome homeostasis genes, including genes that are involved in environmental adaptation extending beyond the ionome, will be those best detected through multivariate methods.

In addition to being a tool for understanding the genetics of multi‐element regulation, principal components also reflected environmental variation. An across‐environment PCA of all lines was used to find variables that describe variation between lines among all 10 environments. The first two across‐environment PCs capture most of the variation in the ionome across 10 different growouts, much of which is environmental. This can be seen in the ability of aPC1 and aPC2 to separate growouts by location and, in some cases, different years within a location. Thus, components from a PCA done across environments can capture the impact of environment on the ionome as a whole.

In our across‐environment analysis, to account for different sets of IBM lines within environments, we tested an approach of projecting loadings from a PCA on a smaller set of lines onto the full data set. The similarity of the PJs and aPCs led us to conclude that the sampling effects of having different subsets of lines in each environment had little effect on the trait covariance estimation. This approach to validate aPCs may be useful in other studies that seek to connect data from disparate experiments and federate data collected by multiple laboratories. The method of deriving traits across environments using a small set of genotypic checks opens up the possibility of using multi‐trait correlations across environments to permit very large scale GxE mapping experiments on data sets not initially intended for this purpose. Retrospective analysis of data, or further data generation from preexisting biological material present in both public and private spheres, is enabled by this approach. For example, multiple association panels have been constructed for trait mapping in maize. Typically, comparison of multi‐trait correlations across different populations is inhibited by our inability to ensure the 1:1 correspondence of traits. By using the subset of lines common to all mapping populations to create a projection, comparable traits could be reflected onto to full datasets for comprehensive genetic evaluation and the loci detected in each panel could then be compared, as we have done here.

Principal component analysis on all environments is a way to find variation resulting from environmental factors that impacts multiple elements, for example weather or soil variables. The weather data available to us for this study were limited to maximum and minimum temperature. Temperature can alter element accumulation by influencing transpiration rate which in turn modulates elemental movement (Barber, Mackay, Kuchenbuch, & Barraclough, 1988; Baxter et al., 2009; Mozafar, Schreiber, & Oertli, 1993). We observed the strongest correlations for aPC1 and aPC2 during the third and fourth quarters of the growing season. Because seed filling occurs in the latter part of the season, temperature during this time could have a pronounced effect on seed elemental composition. However, the lack of striking correlations between environmental components and the projections and aPCs suggests environmental factors other than temperature must be the strongest factors. Information on soil properties provided insight into a potential driver of the environmental variability captured by aPC2, with a strong negative correlation between aPC2 and soil pH. Soil pH alters element availability in soil, and pH differences between locations should result in different kernel ionomes. Although soil element content measurements were not available for this dataset, differences in soil element concentration could also impact element covariation.

Quantitative trait loci were mapped to the aPCs that describe whole‐ionome variation across environments. These loci may encompass genes that pleiotropically affect the ionome in an environmentally responsive manner. The correlation between aPC2 with pH as well as the finding of an aPC2 QTL for Mo exemplifies the possibility of using across‐environment PCA to detect element homeostasis loci that respond to a particular environmental or soil variable and produce a multi‐element phenotype. To the extent that these differences are adaptive, these alleles can contribute to local adaptation to soil environment and nutrient availability. The identification of aPC QTL indicates that the variation captured by aPCs has both environmental and genetic components. Our previous study using single‐element traits found extensive GxE in this dataset through formal tests, so it is not surprising that we see a large environmental component as well as genetic factors contributing to variation in the across‐environment PCs. Experiments with more extensive weather and soil data, or carefully manipulated environmental contrasts, are needed to create models with additional covariates and precisely represent environmental impacts. Considering location and geographical information, such as proximity to industrial sites or distance from the ocean, might add to the predictive ability of such models. This multivariate approach could be especially powerful in studies with extensive and consistent environmental variable recording, such as the “Genomes to Fields” Initiative, where specific environmental variables could be included in QTL models of multi‐element GxE.

## 5 CONCLUSIONS

Here we have shown that treating the ionome as an interrelated set of traits using PCA within environments can identify novel loci. PCA across environments allowed us to derive traits that described both environmental and genetic variation in the ionome.

## CONFLICT OF INTEREST

The authors declare no conflict of interest.

## AUTHORS CONTRIBUTION

AAF conducted all data analysis. AAF interpreted results, created figures, and wrote the manuscript with assistance from BD and IB.
