# Supplementary figures and images for: Multivariate analysis reveals environmental and genetic determinants of element covariation in the maize grain ionome
Source: Plant Direct. 2019 May 10;3(5):e00139. doi: 10.1002/pld3.139 (PMC6589523; doi:10.1002/pld3.139)

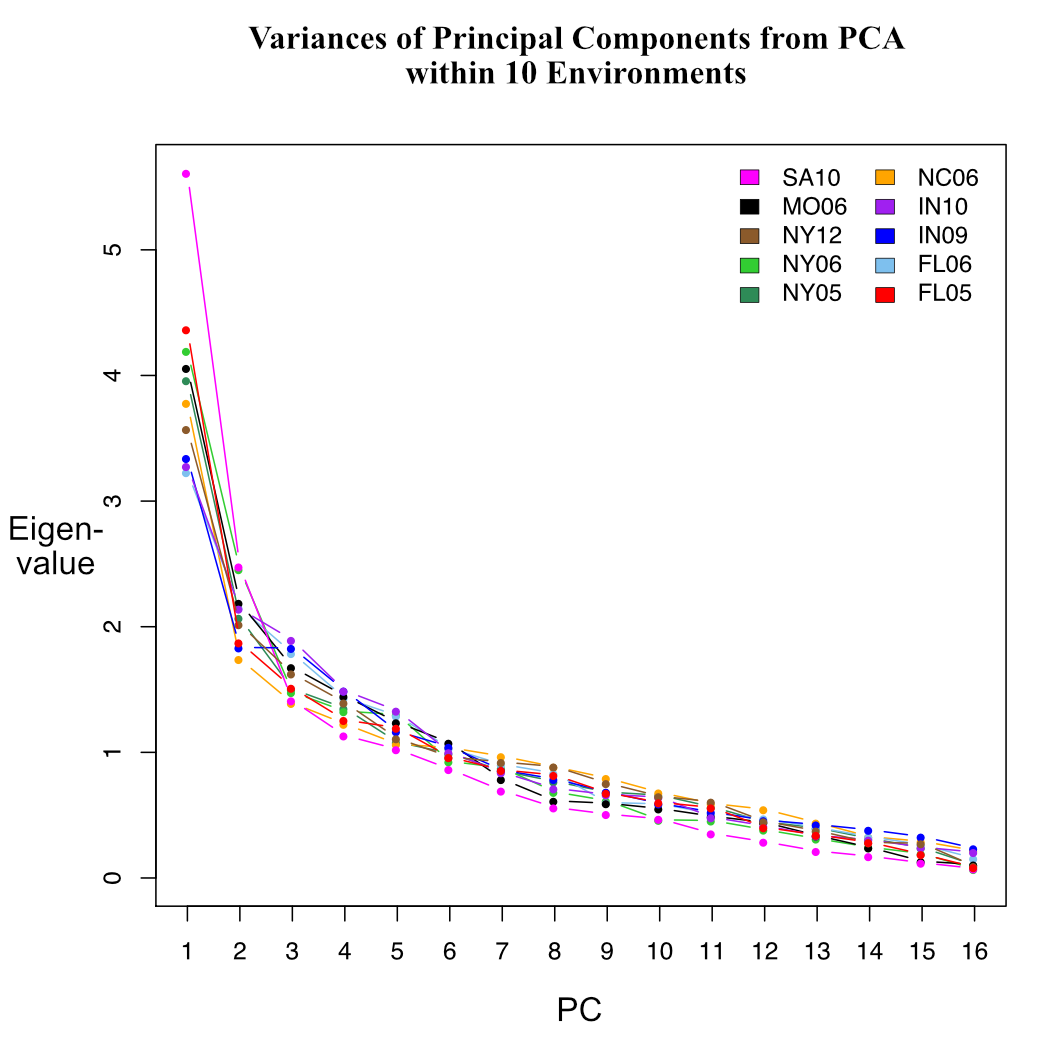

Supplement: Supplementary file 1 [file PLD3-3-e00139-s001.tiff]

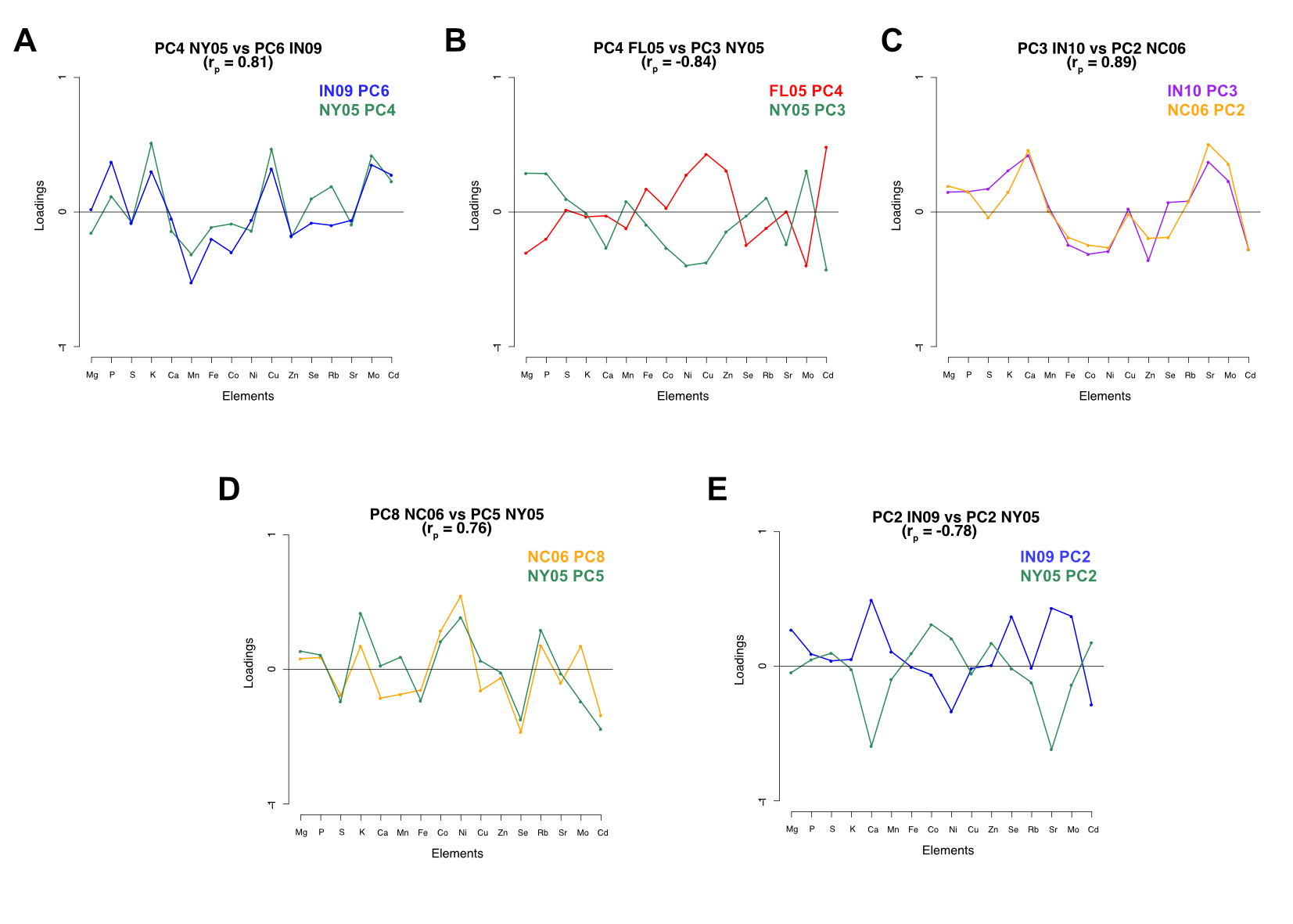

Supplement: Supplementary file 2 [file PLD3-3-e00139-s002.tiff]

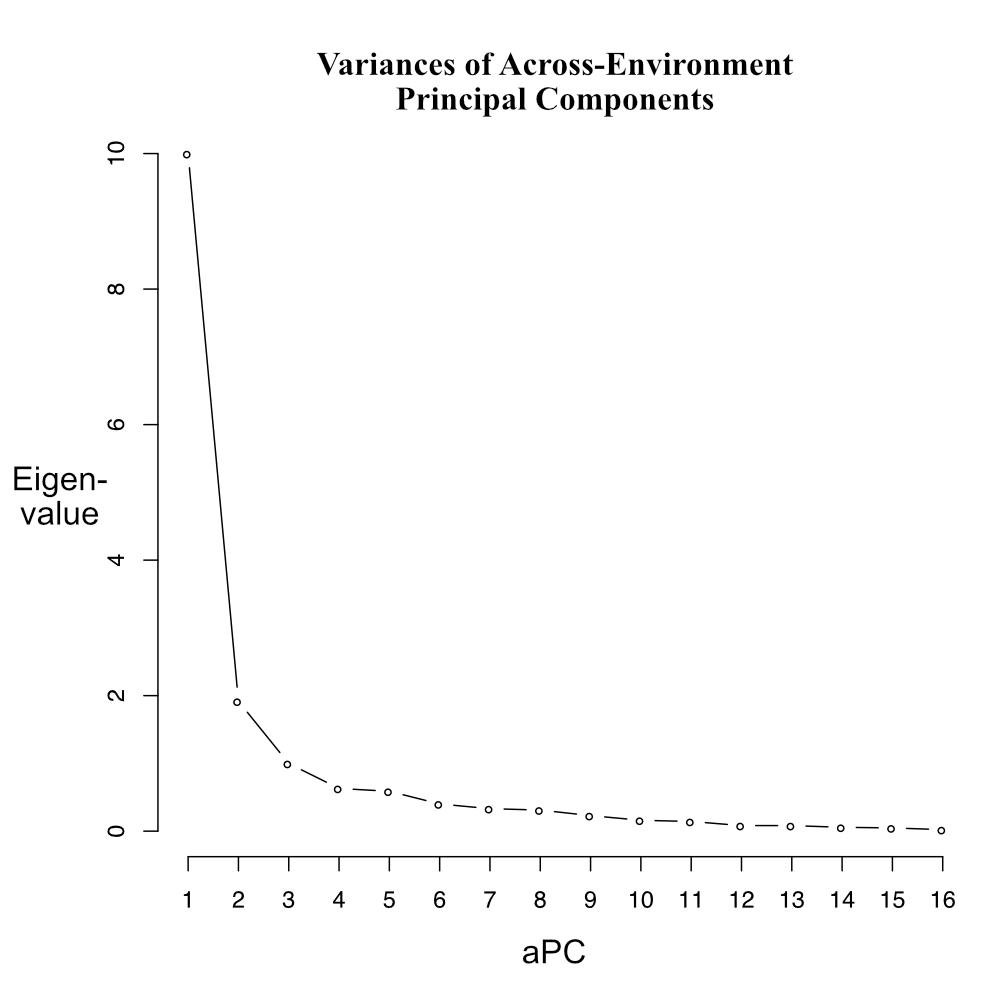

Supplement: Supplementary file 3 [file PLD3-3-e00139-s003.tiff]

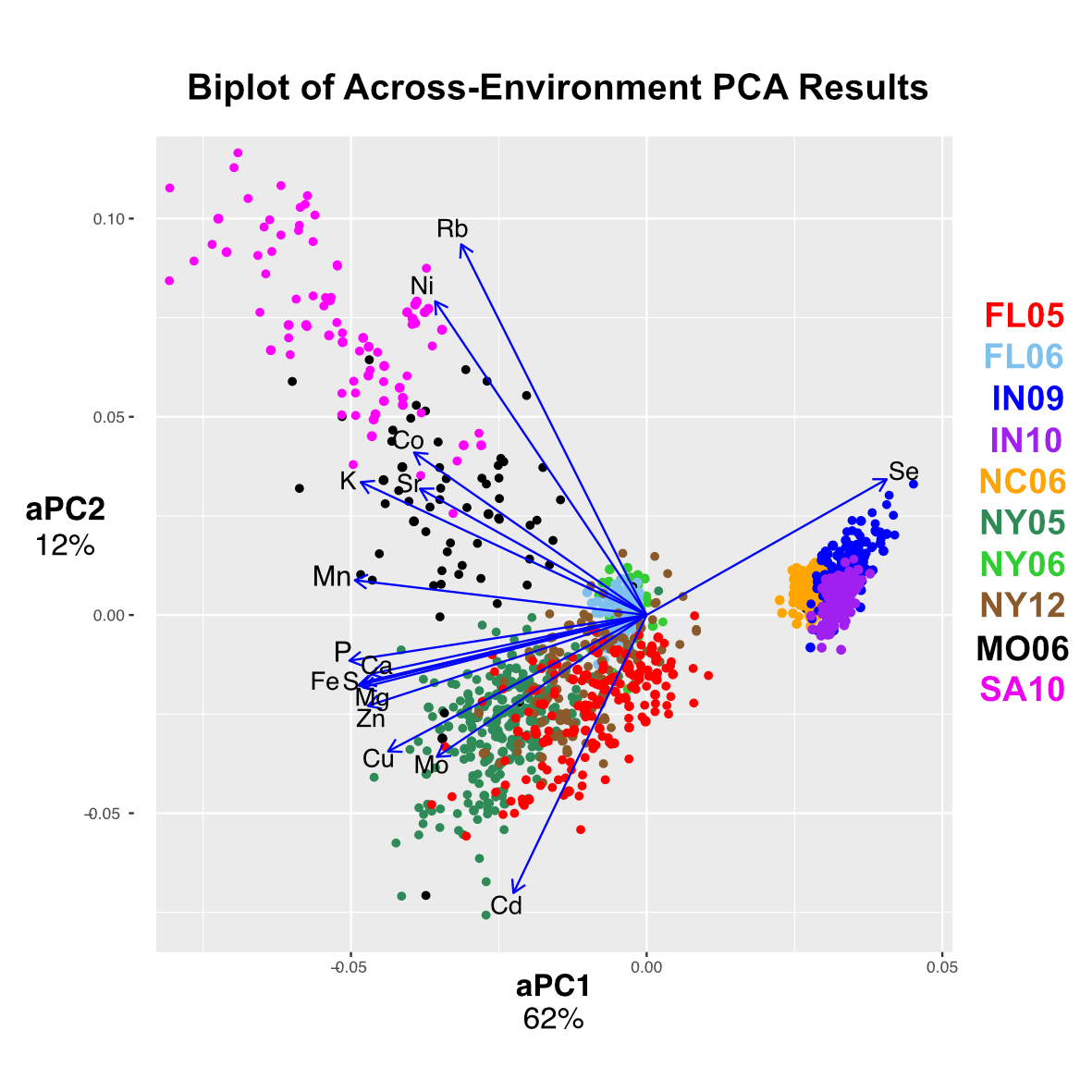

Supplement: Supplementary file 4 [file PLD3-3-e00139-s004.tiff]
