# Supplementary material for: Multivariate analysis reveals environmental and genetic determinants of element covariation in the maize grain ionome
Source: Plant Direct. 2019 May 10;3(5):e00139. doi: 10.1002/pld3.139 (PMC6589523; doi:10.1002/pld3.139)
Supplement: Supplementary file 5 [file PLD3-3-e00139-s005.pdf]

	Overall _Total	FL05	FL06	IN09	IN10	MO06	NC06	NY05	NY06	NY12	SA10
PC1	5	2	0	0	0	1	1	1	0	0	0
PC2	6	0	0	1	0	0	1	4	0	0	0
PC3	10	1	0	0	1	0	0	5	1	2	0
PC4	10	2	0	1	0	0	1	5	0	1	0
PC5	22	1	1	0	1	0	1	6	9	1	2
PC6	9	0	0	1	1	0	2	3	1	1	0
PC7	7	0	0	2	1	0	1	0	0	3	0
PC8	3	0	0	1	1	0	1	0	0	0	0
PC9	6	0	0	1	0	0	1	1	0	1	2
PC10	4	0	0	1	0	0	1	2	0	0	0
PC11	2	0	0	0	0	0	0	2	0	0	0
PC12	6	0	0	2	1	0	3	0	0	0	0
PC13	2	0	0	1	0	0	0	1	0	0	0
PC14	2	0	0	0	1	0	0	0	0	0	1
PC15	4	0	0	0	0	0	1	2	0	0	1
PC16	2	1	0	0	0	0	0	1	0	0	0

Table S1: Within Environment PCs QTL Counts. PC traits calculated separately within environments are listed with QTL number counts overall and within environments.
