# Supplementary material for: Multivariate analysis reveals environmental and genetic determinants of element covariation in the maize grain ionome
Source: Plant Direct. 2019 May 10;3(5):e00139. doi: 10.1002/pld3.139 (PMC6589523; doi:10.1002/pld3.139)
Supplement: Supplementary file 6 [file PLD3-3-e00139-s006.pdf]

Table S2. PC Loadings and Element QTL Overlap

Each PC QTL is shown with the QTL location, environment, LOD score, 95th percentile LOD threshold from 1000 random permutations, percent variance of the QTL, effect magnitude and effect direction. Elements with the top 5 loadings into the PC trait are listed. Unique PC QTL are highlighted (QTL with no overlap within 25 cM). For PC QTL that have element QTL within 10 cM, the element traits for these QTL are listed.

Trait	Chr	Pos	Envirs	LOD	LOD Threshold	%Var	Effect Magnitude	Effect Direction	Elements with top 5 PC loadings (increasing order)	Elemental QTL within 25 cM?	Elements with QTL within 10 cM
PC1	4	173.9	FL05	5.11	3.69	13.37	9.68	Mo17	Mn, Fe, S, Mg	YES	Mn, K
**PC1**	**4**	**287.6**	**FL05**	**5.09**	**3.69**	**13.30**	**9.61**	**B73**	**Mn, Fe, S, M**	**NO**	
PC3	1	404.0	FL05	3.93	3.71	11.59	7.88	B73	Mg, Fe, Mn, K	YES	Mn
PC4	1	252.4	FL05	4.33	3.73	9.22	5.50	B73	Mg, Zn, Mo, (	YES	
PC4	1	380.6	FL05	11.70	3.73	28.07	24.35	B73	Mg, Zn, Mo, (	YES	
PC5	2	215.0	FL05	6.35	3.68	18.03	14.35	Mo17	Sr, Cu, S, Cd,	YES	Cd
PC5	1	378.0	FL06	4.24	3.68	18.77	15.09	Mo17	Fe, P, Mn, Cd	YES	Mo
PC2	2	216.9	IN09	5.92	3.61	18.40	14.79	B73	Ni, Se, Mo, Sr	YES	Cd
PC4	2	203.2	IN09	5.08	3.63	16.01	12.38	B73	Mg, Cd, Mo, S	YES	
PC6	1	378.0	IN09	7.14	3.56	21.77	18.21	Mo17	K, Cu, Mo, P,	YES	Mo
**PC7**	**3**	**358.5**	**IN09**	**3.91**	**3.63**	**11.18**	**7.55**	**B73**	**Cd, S, K, Mn**	**NO**	
PC7	4	300.0	IN09	3.72	3.63	10.60	6.97	B73	Cd, S, K, Mn,	YES	Fe, K
PC8	9	7.7	IN09	8.87	3.62	26.28	22.66	B73	P, K, Cd, Se, I	YES	Ni
**PC9**	**9**	**302.2**	**IN09**	**3.78**	**3.74**	**12.17**	**8.43**	**Mo17**	**Co, Mo, Rb, I**	**NO**	
PC10	2	236.7	IN09	3.78	3.56	12.18	8.62	B73	Co, Cu, Cd, M	YES	Ni
**PC12**	**1**	**136.5**	**IN09**	**4.08**	**3.62**	**11.63**	**8.01**	**Mo17**	**Co, Cu, Zn, F**	**NO**	
**PC12**	**3**	**267.9**	**IN09**	**4.06**	**3.62**	**11.55**	**7.94**	**B73**	**Co, Cu, Zn, F**	**NO**	
**PC13**	**5**	**33.0**	**IN09**	**4.75**	**3.77**	**15.06**	**11.30**	**Mo17**	**K, Rb, Zn, Se**	**NO**	
PC3	1	378.0	IN10	3.60	3.57	12.07	8.50	Mo17	K, Co, Zn, Sr,	YES	Mo
PC5	2	211.7	IN10	5.36	3.65	17.41	13.76	B73	Ni, Fe, Mn, Sr	YES	Mo, Cd
PC6	2	209.5	IN10	4.48	3.68	14.78	11.10	Mo17	S, Ni, Mn, Cd	YES	Mo, Cd
PC7	4	315.8	IN10	4.45	3.75	14.69	10.93	Mo17	Zn, Rb, K, Mg	YES	
PC8	1	377.3	IN10	7.62	3.71	23.76	20.05	B73	Sr, Co, S, Ni,	YES	Mo
**PC12**	**2**	**102.2**	**IN10**	**4.32**	**3.65**	**14.29**	**10.64**	**Mo17**	**Zn, Mn, Cu, (**	**NO**	
PC1	10	95.5	MO06	3.79	3.70	29.48	25.78	Mo17	S, Rb, Fe, K, I	YES	Rb
PC1	7	167.0	NC06	4.62	3.65	13.14	9.49	B73	Rb, Mg, Zn, P	YES	Rb
PC2	1	378.0	NC06	4.47	3.70	12.74	9.04	Mo17	Ni, Cd, Mo, C	YES	Mo
PC4	9	16.8	NC06	4.36	3.58	12.45	8.87	Mo17	Mn, P, Ca, Co	YES	Ni
**PC5**	**3**	**358.7**	**NC06**	**4.48**	**3.50**	**12.77**	**9.27**	**B73**	**K, Mn, Cu, R**	**NO**	
**PC6**	**1**	**244.9**	**NC06**	**4.92**	**3.62**	**12.29**	**8.66**	**B73**	**S, Ni, Mn, Fe**	**NO**	
PC6	2	217.9	NC06	4.11	3.62	10.15	6.52	Mo17	S, Ni, Mn, Fe,	YES	Cd
PC7	2	215.0	NC06	12.03	3.75	30.71	26.96	B73	Fe, S, Ni, Cu,	YES	Cd
PC8	9	8.9	NC06	6.85	3.71	18.86	15.15	B73	Ca, Co, Cd, Se	YES	Ni
**PC9**	**3**	**148.6**	**NC06**	**4.39**	**3.59**	**12.53**	**8.94**	**B73**	**Se, Ni, Cu, Zr**	**NO**	
**PC10**	**3**	**156.8**	**NC06**	**3.91**	**3.62**	**11.23**	**7.62**	**B73**	**Sr, S, Cu, Rb**	**NO**	
**PC12**	**1**	**113.8**	**NC06**	**8.30**	**3.75**	**19.02**	**15.27**	**Mo17**	**Mn, Rb, Fe, (**	**NO**	
**PC12**	**1**	**515.3**	**NC06**	**4.74**	**3.75**	**10.27**	**6.52**	**Mo17**	**Mn, Rb, Fe, (**	**NO**	
**PC12**	**9**	**146.3**	**NC06**	**4.32**	**3.75**	**9.31**	**5.56**	**Mo17**	**Mn, Rb, Fe, (**	**NO**	
PC1	5	203.8	NY05	6.92	3.73	14.46	10.72	Mo17	Mn, Zn, Fe, M	YES	Mn, Fe, Zn, P, S
PC2	2	216.9	NY05	5.57	3.61	8.78	5.17	Mo17	Cd, Ni, Co, Ca	YES	Cd
PC2	3	331.0	NY05	5.48	3.61	8.63	5.02	B73	Cd, Ni, Co, Ca	YES	Sr, Ca
PC2	7	193.8	NY05	4.63	3.61	7.21	3.60	B73	Cd, Ni, Co, Ca	YES	Sr
PC2	9	0.9	NY05	6.34	3.61	10.08	6.47	B73	Cd, Ni, Co, Ca	YES	Ni

PC3	1	377.3	NY05	14.78	3.78	19.39	15.61	Mo17	Mg, Mo, Cu,	YES	Mo
PC3	2	211.0	NY05	12.89	3.78	16.53	12.75	B73	Mg, Mo, Cu,	YES	Cd
PC3	9	5.4	NY05	5.66	3.78	6.67	2.89	Mo17	Mg, Mo, Cu,	YES	Ni
**PC3**	**10**	**87.8**	**NY05**	**4.63**	**3.78**	**5.40**	**1.62**	**Mo17**	**Mg, Mo, Cu,**	**NO**	
**PC3**	**10**	**121.6**	**NY05**	**7.30**	**3.78**	**8.76**	**4.99**	**B73**	**Mg, Mo, Cu,**	**NO**	
PC4	1	378.0	NY05	8.49	3.55	12.49	8.94	Mo17	Cd, Mn, Mo,	YES	Mo
PC4	2	218.3	NY05	3.98	3.55	5.56	2.01	Mo17	Cd, Mn, Mo,	YES	Cd
PC4	3	325.8	NY05	4.18	3.55	5.84	2.29	B73	Cd, Mn, Mo,	YES	Sr, Ca
PC4	4	178.9	NY05	4.08	3.55	5.69	2.15	Mo17	Cd, Mn, Mo,	YES	K
PC4	7	165.9	NY05	7.54	3.55	10.97	7.42	Mo17	Cd, Mn, Mo,	YES	Cu
PC5	1	171.4	NY05	5.20	3.68	6.75	3.07	B73	Rb, Se, Ni, K,	YES	K
PC5	2	208.9	NY05	7.62	3.68	10.17	6.49	B73	Rb, Se, Ni, K,	YES	Cd
PC5	4	374.9	NY05	6.67	3.68	8.81	5.13	B73	Rb, Se, Ni, K,	YES	
PC5	7	150.7	NY05	3.96	3.68	5.06	1.39	Mo17	Rb, Se, Ni, K,	YES	K
PC5	9	7.7	NY05	8.48	3.68	11.43	7.75	B73	Rb, Se, Ni, K,	YES	Ni
PC5	9	136.6	NY05	4.83	3.68	6.25	2.57	Mo17	Rb, Se, Ni, K,	YES	Ni
PC6	1	378.0	NY05	14.86	3.58	23.71	20.13	Mo17	K, Cd, Rb, Ni	YES	Mo
PC6	2	214.6	NY05	5.77	3.58	8.28	4.70	Mo17	K, Cd, Rb, Ni	YES	Cd
PC6	9	8.3	NY05	5.00	3.58	7.11	3.53	B73	K, Cd, Rb, Ni	YES	Ni
PC9	9	5.4	NY05	4.09	3.72	8.82	5.10	B73	Cd, Zn, Ni, C	YES	Ni
PC10	1	385.7	NY05	4.33	3.60	8.62	5.02	B73	Mn, Zn, Mo,	YES	Mo
**PC10**	**10**	**147.6**	**NY05**	**4.12**	**3.60**	**8.17**	**4.57**	**B73**	**Mn, Zn, Mo,**	**NO**	
**PC11**	**6**	**128.6**	**NY05**	**4.69**	**3.72**	**9.42**	**5.70**	**Mo17**	**Mg, Mn, K,**	**NO**	
PC11	6	256.4	NY05	4.73	3.72	9.50	5.78	Mo17	Mg, Mn, K, C	YES	
PC13	1	232.0	NY05	4.07	3.63	8.77	5.15	Mo17	K, Mo, P, Mg	YES	Mn
**PC3**	**6**	**42.5**	**NY06**	**3.64**	**3.60**	**30.57**	**26.97**	**Mo17**	**Cd, Cu, Rb,**	**NO**	
**PC5**	**1**	**167.0**	**NY06**	**3.71**	**3.03**	**1.43**	**1.60**	**Mo17**	**Mn, Mg, Co,**	**NO**	
**PC5**	**1**	**169.7**	**NY06**	**14.43**	**3.03**	**10.30**	**7.28**	**Mo17**	**Mn, Mg, Co,**	**NO**	
**PC5**	**1**	**271.2**	**NY06**	**21.58**	**3.03**	**24.41**	**21.38**	**B73**	**Mn, Mg, Co,**	**NO**	
PC5	1	379.7	NY06	19.95	3.03	20.25	17.22	B73	Mn, Mg, Co,	YES	Mo
PC5	2	98.3	NY06	14.33	3.03	10.17	7.14	B73	Mn, Mg, Co,	YES	Mo
**PC5**	**2**	**257.5**	**NY06**	**10.76**	**3.03**	**6.16**	**3.13**	**B73**	**Mn, Mg, Co,**	**NO**	
**PC5**	**4**	**75.9**	**NY06**	**4.43**	**3.03**	**1.78**	**1.25**	**B73**	**Mn, Mg, Co,**	**NO**	
**PC5**	**6**	**109.3**	**NY06**	**8.99**	**3.03**	**4.64**	**1.61**	**B73**	**Mn, Mg, Co,**	**NO**	
**PC5**	**6**	**158.0**	**NY06**	**16.68**	**3.03**	**13.70**	**10.68**	**B73**	**Mn, Mg, Co,**	**NO**	
**PC5**	**8**	**355.7**	**NY06**	**6.57**	**3.03**	**2.96**	**0.07**	**B73**	**Mn, Mg, Co,**	**NO**	
**PC6**	**7**	**162.3**	**NY06**	**4.33**	**3.66**	**35.20**	**31.55**	**Mo17**	**Mo, Sr, Mn,**	**NO**	
PC3	2	214.1	NY12	5.48	3.65	19.23	15.59	B73	S, Rb, Ni, K,	YES	Cd
PC3	9	0.0	NY12	3.68	3.65	12.40	8.75	Mo17	S, Rb, Ni, K,	YES	Ni
**PC4**	**3**	**221.5**	**NY12**	**3.72**	**3.70**	**15.21**	**11.50**	**B73**	**Mn, Se, Co, K**	**NO**	
**PC5**	**5**	**150.9**	**NY12**	**3.59**	**3.56**	**14.71**	**11.14**	**B73**	**Fe, Se, Mo, K**	**NO**	
PC6	2	210.8	NY12	3.72	3.59	15.19	11.60	B73	Mg, Zn, Cd, M	YES	Cd
PC7	2	242.5	NY12	4.62	3.58	13.90	10.31	B73	Rb, Cd, S, Mg	YES	Ni
**PC7**	**5**	**107.0**	**NY12**	**4.72**	**3.58**	**14.24**	**10.65**	**B73**	**Rb, Cd, S, M**	**NO**	
**PC7**	**6**	**255.4**	**NY12**	**4.07**	**3.58**	**12.11**	**8.53**	**Mo17**	**Rb, Cd, S, M**	**NO**	
**PC9**	**1**	**342.2**	**NY12**	**5.12**	**3.69**	**20.28**	**16.59**	**Mo17**	**Zn, Mo, Se, R**	**NO**	
**PC5**	**1**	**83.5**	**SA10**	**4.65**	**3.68**	**22.20**	**18.52**	**B73**	**Co, Cd, Mg,**	**NO**	
**PC5**	**4**	**382.9**	**SA10**	**3.83**	**3.68**	**17.76**	**14.09**	**B73**	**Co, Cd, Mg,**	**NO**	
**PC9**	**1**	**418.2**	**SA10**	**4.77**	**3.28**	**22.26**	**18.99**	**Mo17**	**Fe, Ni, Co, R**	**NO**	
**PC9**	**7**	**169.8**	**SA10**	**4.25**	**3.28**	**19.46**	**16.19**	**B73**	**Fe, Ni, Co, R**	**NO**	
