# Supplementary material for: Multivariate analysis reveals environmental and genetic determinants of element covariation in the maize grain ionome
Source: Plant Direct. 2019 May 10;3(5):e00139. doi: 10.1002/pld3.139 (PMC6589523; doi:10.1002/pld3.139)
Supplement: Supplementary file 7 [file PLD3-3-e00139-s007.pdf]

Table S3. PC Variance Proportions and Loadings Across 10 Environments.

Loadings of elements into each PC within environments.

FL05	PC1	PC2	PC3	PC4	PC5	PC6	PC7	PC8	PC9	PC10	PC11	PC12	PC13	PC14	PC15	PC16
Standard deviation	2.09	1.37	1.23	1.12	1.09	0.98	0.93	0.91	0.82	0.78	0.75	0.64	0.59	0.53	0.44	0.29
Proportion of Variance	0.27	0.12	0.09	0.08	0.07	0.06	0.05	0.05	0.04	0.04	0.04	0.03	0.02	0.02	0.01	0.01
Cumulative Proportion	0.27	0.39	0.48	0.56	0.64	0.70	0.75	0.80	0.85	0.88	0.92	0.94	0.96	0.98	0.99	1.00
Mg	0.33	-0.03	0.20	-0.30	0.09	-0.24	0.07	0.13	-0.42	0.11	0.08	0.41	-0.15	-0.18	0.50	-0.11
P	0.38	-0.13	-0.04	-0.20	0.11	0.00	0.12	0.26	-0.27	0.20	-0.02	0.13	0.18	0.21	-0.69	0.14
S	0.33	0.01	0.12	0.02	0.36	-0.15	0.05	-0.10	0.15	0.24	0.40	-0.57	-0.37	0.04	0.02	0.08
K	0.24	0.01	-0.56	-0.04	-0.05	-0.20	0.32	-0.13	-0.01	-0.05	-0.14	-0.17	0.28	0.47	0.34	-0.03
Ca	0.26	0.53	0.12	-0.03	-0.20	-0.04	-0.07	-0.11	0.10	-0.11	0.09	0.05	-0.03	0.13	-0.21	-0.69
Mn	0.28	-0.05	0.33	-0.12	-0.07	0.22	0.26	-0.11	-0.19	-0.50	-0.38	-0.41	0.07	-0.23	0.00	0.06
Fe	0.32	-0.18	0.29	0.17	0.02	0.08	-0.01	0.00	0.43	0.20	0.13	0.09	0.66	-0.16	0.16	-0.03
Co	-0.05	-0.33	0.00	0.03	-0.63	0.01	0.17	-0.33	-0.28	0.07	0.50	-0.08	0.02	-0.07	-0.09	-0.03
Ni	0.17	-0.17	-0.09	0.28	-0.17	-0.54	-0.44	0.39	-0.05	-0.39	0.07	-0.15	0.05	-0.08	-0.04	0.02
Cu	0.23	0.10	-0.15	0.43	-0.30	0.21	-0.07	0.19	-0.18	0.52	-0.37	-0.17	-0.17	-0.20	0.09	-0.06
Zn	0.26	-0.31	0.05	0.31	-0.11	0.13	0.30	0.09	0.37	-0.24	-0.03	0.38	-0.47	0.22	-0.01	0.02
Se	0.16	-0.32	0.08	-0.25	-0.09	-0.16	-0.49	-0.53	0.13	0.16	-0.39	0.05	-0.13	0.15	-0.05	0.00
Rb	0.23	-0.01	-0.59	-0.12	0.14	0.05	0.02	-0.20	0.17	-0.13	0.07	0.14	-0.06	-0.65	-0.15	-0.05
Sr	0.23	0.56	0.06	0.00	-0.28	-0.07	-0.07	-0.16	0.08	-0.05	0.08	0.17	-0.01	0.02	0.02	0.69
Mo	0.16	-0.07	-0.17	-0.40	-0.16	0.59	-0.39	0.32	0.08	-0.10	0.23	-0.12	-0.04	0.18	0.21	0.02
Cd	0.15	0.00	-0.05	0.48	0.38	0.31	-0.28	-0.34	-0.43	-0.19	0.16	0.15	0.09	0.17	0.04	0.00

FL06	PC1	PC2	PC3	PC4	PC5	PC6	PC7	PC8	PC9	PC10	PC11	PC12	PC13	PC14	PC15	PC16
Standard deviation	1.80	1.47	1.34	1.20	1.14	1.02	0.95	0.91	0.77	0.77	0.72	0.69	0.63	0.57	0.52	0.40
Proportion of Variance	0.20	0.13	0.11	0.09	0.08	0.06	0.06	0.05	0.04	0.04	0.03	0.03	0.03	0.02	0.02	0.01
Cumulative Proportion	0.20	0.34	0.45	0.54	0.62	0.68	0.74	0.79	0.83	0.87	0.90	0.93	0.95	0.97	0.99	1.00
Mg	0.36	-0.23	0.23	-0.05	0.04	-0.34	0.05	-0.03	-0.12	0.41	0.14	-0.15	-0.02	0.25	-0.55	-0.23
P	0.42	-0.04	0.10	-0.18	0.23	-0.10	-0.21	-0.22	-0.06	0.20	-0.08	-0.38	0.23	0.04	0.58	0.21
S	0.42	-0.11	0.09	-0.10	0.11	0.04	0.16	0.22	0.30	0.22	0.00	0.38	-0.40	-0.50	0.11	0.08
K	0.32	0.24	0.08	-0.18	0.02	0.47	-0.07	0.01	0.23	-0.25	0.53	-0.09	-0.01	0.20	0.02	-0.35
Ca	0.22	0.03	-0.52	0.19	-0.21	-0.15	-0.04	0.33	0.15	0.15	-0.25	0.07	0.18	0.16	0.23	-0.50
Mn	0.35	0.06	0.12	0.09	-0.27	-0.16	0.40	-0.11	-0.41	-0.42	0.01	0.03	0.29	-0.37	0.02	-0.13
Fe	0.21	0.03	0.24	0.43	0.22	0.03	-0.19	-0.44	0.31	-0.24	-0.38	0.28	0.04	0.14	-0.12	-0.09

Co	-0.11	0.24	0.18	0.54	-0.03	0.23	-0.21	-0.01	-0.23	0.50	0.26	0.05	0.17	-0.29	0.07	-0.11
Ni	0.03	0.50	-0.05	0.09	0.02	-0.46	0.01	-0.17	-0.20	-0.04	0.19	0.13	-0.55	0.22	0.23	-0.02
Cu	0.19	0.15	-0.40	-0.02	0.11	0.48	0.16	-0.26	-0.33	0.14	-0.35	-0.22	-0.31	-0.05	-0.22	-0.01
Zn	0.28	-0.11	-0.13	0.52	-0.03	0.00	-0.13	0.39	0.02	-0.27	0.14	-0.34	-0.16	0.07	-0.12	0.44
Se	0.04	-0.53	0.06	0.07	0.05	0.24	-0.06	0.07	-0.49	-0.07	0.08	0.41	-0.14	0.34	0.27	-0.05
Rb	0.19	0.46	0.19	-0.16	-0.12	0.14	0.08	0.32	-0.11	0.14	-0.22	0.33	0.27	0.35	-0.11	0.39
Sr	0.17	-0.08	-0.51	-0.09	-0.21	-0.07	-0.25	-0.39	0.04	0.07	0.34	0.34	0.21	-0.06	-0.18	0.32
Mo	0.02	0.15	-0.15	-0.15	0.64	-0.15	-0.38	0.28	-0.27	-0.21	0.00	0.13	0.15	-0.23	-0.20	-0.13
Cd	-0.07	-0.01	-0.21	0.22	0.53	-0.02	0.65	-0.05	0.14	0.10	0.24	0.07	0.24	0.17	0.08	0.10

IN09	PC1	PC2	PC3	PC4	PC5	PC6	PC7	PC8	PC9	PC10	PC11	PC12	PC13	PC14	PC15	PC16
Standard deviation	1.83	1.35	1.35	1.22	1.08	1.02	0.93	0.89	0.83	0.77	0.73	0.68	0.65	0.62	0.57	0.49
Proportion of Variance	0.21	0.11	0.11	0.09	0.07	0.07	0.05	0.05	0.04	0.04	0.03	0.03	0.03	0.02	0.02	0.01
Cumulative Proportion	0.21	0.32	0.44	0.53	0.60	0.67	0.72	0.77	0.82	0.85	0.89	0.91	0.94	0.96	0.99	1.00
Mg	-0.27	0.27	-0.21	0.25	-0.04	0.02	-0.49	0.00	-0.16	0.10	0.50	0.17	0.29	0.10	0.31	0.07
P	-0.28	0.09	-0.34	0.19	-0.09	0.37	-0.07	0.18	-0.01	0.20	-0.32	-0.57	-0.12	-0.30	0.09	-0.05
S	-0.34	0.04	-0.08	0.19	0.17	-0.08	0.32	-0.08	-0.48	-0.57	0.04	-0.03	0.13	-0.22	-0.20	0.16
K	-0.34	0.05	0.11	0.02	0.36	0.30	0.35	0.19	-0.13	0.20	0.16	0.13	-0.32	0.44	0.07	-0.29
Ca	-0.02	0.49	0.44	-0.03	-0.13	-0.05	0.07	0.07	-0.13	0.19	-0.05	-0.17	-0.15	0.11	-0.03	0.64
Mn	-0.31	0.11	-0.08	0.08	0.07	-0.52	-0.39	-0.07	-0.09	0.05	-0.46	0.09	-0.24	0.25	-0.25	-0.16
Fe	-0.36	-0.01	-0.08	-0.16	-0.24	-0.20	0.22	-0.10	0.46	-0.11	0.24	-0.42	0.26	0.36	-0.15	-0.03
Co	-0.06	-0.06	-0.02	-0.62	0.24	-0.30	-0.09	0.08	-0.31	0.24	0.32	-0.28	-0.08	-0.30	-0.02	-0.10
Ni	-0.16	-0.34	0.20	0.05	-0.13	-0.06	-0.18	0.83	0.08	-0.15	0.06	0.08	0.03	-0.04	-0.14	0.11
Cu	-0.36	-0.02	0.19	-0.22	-0.14	0.32	-0.02	-0.16	-0.03	0.33	-0.16	0.34	0.41	-0.20	-0.42	-0.05
Zn	-0.40	0.01	-0.08	-0.16	-0.23	-0.18	0.21	-0.07	0.29	-0.04	-0.03	0.38	-0.35	-0.36	0.44	0.12
Se	0.13	0.37	-0.29	-0.35	-0.04	-0.08	0.22	0.36	-0.13	-0.07	-0.36	0.14	0.41	0.18	0.27	-0.09
Rb	-0.13	-0.01	0.31	0.10	0.68	-0.10	-0.06	-0.02	0.35	0.01	-0.20	-0.10	0.34	-0.15	0.28	0.08
Sr	0.03	0.43	0.48	0.12	-0.23	-0.06	-0.03	0.05	0.03	-0.20	0.08	-0.10	-0.01	-0.25	0.03	-0.62
Mo	0.02	0.37	-0.18	-0.34	0.26	0.35	-0.30	0.04	0.33	-0.41	0.08	0.09	-0.24	-0.03	-0.28	0.09
Cd	-0.16	-0.28	0.30	-0.33	-0.16	0.28	-0.30	-0.20	-0.24	-0.35	-0.21	-0.15	-0.01	0.26	0.38	0.01

IN10	PC1	PC2	PC3	PC4	PC5	PC6	PC7	PC8	PC9	PC10	PC11	PC12	PC13	PC14	PC15	PC16
Standard deviation	1.81	1.46	1.38	1.22	1.15	1.00	0.92	0.84	0.82	0.80	0.70	0.64	0.58	0.55	0.49	0.45
Proportion of Variance	0.20	0.13	0.12	0.09	0.08	0.06	0.05	0.04	0.04	0.04	0.03	0.03	0.02	0.02	0.02	0.01
Cumulative Proportion	0.20	0.34	0.46	0.55	0.63	0.70	0.75	0.79	0.84	0.88	0.91	0.93	0.95	0.97	0.99	1.00
Mg	-0.29	-0.04	0.15	-0.40	0.09	-0.23	0.50	-0.07	0.20	-0.20	0.23	-0.06	-0.13	0.49	-0.02	0.15

P	-0.35	0.30	0.15	-0.18	0.03	0.01	0.09	-0.15	-0.10	-0.44	-0.22	-0.19	0.50	-0.35	0.11	-0.20
S	-0.36	0.07	0.17	-0.15	0.22	0.28	-0.06	0.38	0.33	0.25	0.10	-0.30	-0.33	-0.33	-0.17	-0.14
K	-0.27	0.18	0.31	0.22	0.23	0.22	-0.43	-0.07	-0.09	0.19	0.13	-0.13	0.29	0.48	0.13	0.22
Ca	-0.10	-0.40	0.42	0.18	-0.25	-0.03	0.07	0.02	-0.02	-0.08	-0.29	-0.12	0.04	-0.17	-0.36	0.53
Mn	-0.27	-0.32	0.04	-0.06	0.31	-0.37	0.01	-0.18	-0.06	0.39	0.27	0.37	0.24	-0.35	0.07	0.04
Fe	-0.37	-0.01	-0.24	-0.17	-0.30	-0.24	-0.21	0.11	-0.11	0.12	-0.29	-0.13	-0.27	-0.02	0.56	0.26
Co	-0.22	-0.07	-0.31	0.38	-0.23	-0.27	-0.15	-0.27	0.15	-0.16	0.45	-0.43	-0.03	-0.06	-0.20	-0.09
Ni	-0.10	-0.29	-0.29	0.18	0.28	0.31	0.13	-0.39	0.50	0.01	-0.40	-0.01	0.04	0.06	0.16	0.01
Cu	-0.37	-0.06	0.02	0.26	0.13	0.25	-0.04	-0.08	-0.34	-0.43	0.08	0.43	-0.46	-0.04	-0.01	-0.05
Zn	-0.33	-0.06	-0.36	-0.22	-0.18	0.00	-0.26	0.22	0.06	0.02	-0.22	0.30	0.25	0.25	-0.52	-0.15
Se	-0.05	0.46	0.07	0.28	-0.22	-0.10	0.05	0.16	0.55	-0.08	0.10	0.45	0.07	-0.12	0.11	0.26
Rb	-0.14	0.31	0.08	0.41	0.16	-0.41	0.28	-0.01	-0.15	0.29	-0.42	-0.06	-0.16	0.14	-0.18	-0.25
Sr	-0.07	-0.42	0.37	0.19	-0.31	-0.04	0.00	0.24	0.17	-0.04	0.01	0.06	0.11	0.17	0.32	-0.56
Mo	-0.08	0.18	0.23	-0.21	-0.49	0.24	0.02	-0.60	0.00	0.35	0.02	0.12	-0.17	-0.02	-0.10	-0.16
Cd	-0.19	-0.01	-0.28	0.21	-0.22	0.40	0.56	0.23	-0.27	0.26	0.19	-0.05	0.26	0.00	0.09	0.10

NC06	PC1	PC2	PC3	PC4	PC5	PC6	PC7	PC8	PC9	PC10	PC11	PC12	PC13	PC14	PC15	PC16
Standard deviation	1.94	1.32	1.18	1.11	1.03	1.02	0.98	0.94	0.89	0.83	0.77	0.74	0.66	0.57	0.54	0.47
Proportion of Variance	0.24	0.11	0.09	0.08	0.07	0.06	0.06	0.06	0.05	0.04	0.04	0.03	0.03	0.02	0.02	0.01
Cumulative Proportion	0.24	0.35	0.43	0.51	0.58	0.64	0.70	0.76	0.81	0.85	0.89	0.92	0.95	0.97	0.99	1.00
Mg	-0.34	0.19	-0.06	0.12	-0.51	0.14	-0.04	0.08	-0.11	0.05	-0.06	0.19	0.06	-0.23	-0.60	-0.26
P	-0.37	0.15	-0.09	0.30	-0.22	-0.03	0.12	0.09	-0.29	0.25	0.00	0.17	-0.15	-0.12	0.45	0.51
S	-0.32	-0.04	-0.22	-0.12	0.14	-0.27	-0.22	-0.19	-0.13	0.31	0.11	-0.65	-0.22	-0.03	-0.23	0.06
K	-0.38	0.15	-0.22	-0.10	0.28	-0.15	0.03	0.17	-0.17	0.10	-0.02	0.19	0.10	0.22	0.32	-0.63
Ca	-0.06	0.46	0.40	-0.30	-0.05	0.01	-0.07	-0.21	0.07	-0.13	0.40	0.11	-0.50	-0.10	0.11	-0.11
Mn	-0.25	0.01	0.19	0.24	-0.32	-0.41	-0.12	-0.18	0.04	-0.59	-0.06	-0.23	0.22	0.21	0.16	-0.04
Fe	-0.31	-0.19	0.07	0.09	-0.03	0.41	0.21	-0.15	0.43	0.06	0.10	-0.29	0.17	-0.41	0.29	-0.20
Co	-0.17	-0.25	0.10	-0.55	-0.23	-0.12	-0.03	0.29	0.18	-0.03	-0.56	0.01	-0.28	-0.10	0.12	0.05
Ni	-0.12	-0.27	0.37	-0.09	-0.02	0.29	-0.24	0.55	-0.34	-0.08	0.36	-0.20	0.12	0.12	0.01	0.03
Cu	-0.23	-0.02	0.24	0.13	0.43	0.26	0.36	-0.16	-0.36	-0.31	-0.38	-0.07	-0.24	-0.01	-0.20	0.03
Zn	-0.34	-0.20	0.25	0.05	0.04	-0.03	0.21	-0.06	0.41	0.27	0.13	0.21	-0.05	0.59	-0.23	0.16
Se	-0.10	-0.19	-0.23	-0.55	-0.19	0.19	0.16	-0.47	-0.32	-0.11	0.17	0.13	0.28	0.13	0.04	0.15
Rb	-0.32	0.08	-0.15	-0.15	0.43	-0.17	-0.15	0.18	0.23	-0.32	0.17	0.26	0.24	-0.34	-0.19	0.34
Sr	-0.03	0.50	0.37	-0.17	0.09	0.11	-0.20	-0.10	0.00	0.31	-0.35	-0.14	0.50	0.07	0.03	0.15
Mo	-0.06	0.36	-0.46	-0.01	-0.06	0.48	-0.10	0.17	0.23	-0.28	-0.07	-0.22	-0.16	0.39	0.03	0.13
Cd	-0.11	-0.28	0.00	0.18	0.10	0.25	-0.74	-0.34	0.00	0.03	-0.17	0.30	-0.15	0.02	0.09	-0.05

NY05	PC1	PC2	PC3	PC4	PC5	PC6	PC7	PC8	PC9	PC10	PC11	PC12	PC13	PC14	PC15	PC16

Standard deviation	1.99	1.44	1.23	1.16	1.04	1.00	0.92	0.88	0.83	0.81	0.75	0.67	0.63	0.56	0.49	0.32
Proportion of Variance	0.25	0.13	0.09	0.08	0.07	0.06	0.05	0.05	0.04	0.04	0.04	0.03	0.02	0.02	0.02	0.01
Cumulative Proportion	0.25	0.38	0.47	0.56	0.62	0.69	0.74	0.79	0.83	0.87	0.91	0.93	0.96	0.98	0.99	1.00
Mg	0.36	-0.05	0.29	-0.15	0.14	0.14	-0.21	0.18	-0.02	0.07	0.27	-0.26	-0.41	-0.09	-0.56	0.10
P	0.39	0.05	0.29	0.12	0.11	-0.05	-0.22	0.09	0.00	0.18	0.05	-0.03	-0.28	0.24	0.71	0.01
S	0.34	0.10	0.10	-0.08	-0.24	0.03	0.08	-0.33	-0.18	0.25	-0.55	0.46	-0.21	0.00	-0.20	-0.03
K	0.19	-0.02	-0.01	0.51	0.42	-0.31	-0.10	-0.21	0.06	0.15	-0.34	-0.37	0.25	-0.05	-0.18	-0.02
Ca	0.12	-0.59	-0.27	-0.14	0.03	0.04	-0.05	-0.04	-0.05	0.00	-0.14	-0.03	0.01	-0.13	0.14	0.69
Mn	0.35	-0.10	0.08	-0.32	0.09	-0.06	-0.11	0.03	-0.17	0.26	0.28	0.21	0.70	0.09	-0.05	-0.11
Fe	0.36	0.10	-0.10	-0.11	-0.23	0.04	0.17	-0.18	-0.01	-0.51	-0.07	-0.34	0.11	0.57	-0.06	0.06
Co	0.17	0.31	-0.27	-0.09	0.21	-0.06	0.15	0.55	-0.53	-0.17	-0.24	-0.06	-0.01	-0.21	0.07	0.00
Ni	0.05	0.21	-0.40	-0.14	0.39	0.46	-0.24	0.12	0.47	0.07	-0.20	0.17	0.01	0.22	-0.05	-0.01
Cu	0.21	-0.05	-0.38	0.47	0.06	-0.22	-0.07	-0.02	-0.09	-0.21	0.41	0.50	-0.18	0.13	-0.15	0.03
Zn	0.36	0.17	-0.15	-0.18	-0.02	-0.05	-0.05	-0.34	0.27	-0.30	0.13	-0.05	0.00	-0.65	0.20	-0.15
Se	0.25	-0.02	-0.03	0.10	-0.37	-0.28	0.31	0.51	0.55	0.18	-0.07	0.02	0.08	-0.05	-0.06	0.07
Rb	0.15	-0.12	0.10	0.19	0.29	0.46	0.75	-0.10	-0.01	0.09	0.17	0.02	0.00	-0.07	0.07	-0.04
Sr	0.08	-0.62	-0.24	-0.09	-0.03	0.01	-0.04	0.10	-0.05	-0.01	-0.14	-0.11	-0.17	0.04	0.02	-0.68
Mo	0.09	-0.14	0.31	0.42	-0.24	0.48	-0.30	0.23	-0.03	-0.35	-0.17	0.13	0.28	-0.18	-0.01	-0.03
Cd	0.07	0.18	-0.43	0.23	-0.44	0.31	-0.11	-0.09	-0.20	0.47	0.19	-0.34	0.02	-0.06	0.03	0.00

NY06	PC1	PC2	PC3	PC4	PC5	PC6	PC7	PC8	PC9	PC10	PC11	PC12	PC13	PC14	PC15	PC16
Standard deviation	2.05	1.57	1.22	1.15	1.14	0.96	0.94	0.83	0.78	0.68	0.68	0.62	0.56	0.50	0.44	0.27
Proportion of Variance	0.26	0.15	0.09	0.08	0.08	0.06	0.05	0.04	0.04	0.03	0.03	0.02	0.02	0.02	0.01	0.00
Cumulative Proportion	0.26	0.42	0.51	0.59	0.67	0.73	0.79	0.83	0.87	0.90	0.92	0.95	0.97	0.98	1.00	1.00
Mg	-0.37	0.16	-0.08	0.06	-0.33	0.11	-0.24	0.08	-0.03	0.12	0.11	-0.37	0.00	0.51	0.25	-0.39
P	-0.40	0.05	-0.03	0.24	-0.17	-0.14	0.28	-0.14	-0.12	0.03	-0.08	-0.16	-0.34	-0.14	0.38	0.55
S	-0.42	0.13	-0.17	-0.02	0.05	-0.04	0.00	-0.18	-0.05	-0.05	-0.07	-0.16	-0.37	-0.34	-0.60	-0.31
K	-0.30	-0.13	0.13	0.23	0.01	-0.45	0.19	0.29	0.31	-0.07	-0.51	0.16	0.24	0.07	0.00	-0.22
Ca	-0.11	-0.16	-0.27	-0.64	0.09	0.17	-0.07	-0.15	0.23	0.01	-0.47	-0.24	0.11	0.10	0.05	0.23
Mn	-0.29	0.06	-0.11	-0.24	-0.26	0.27	0.42	0.28	0.08	-0.39	0.35	0.08	0.34	-0.21	0.00	0.00
Fe	-0.34	-0.08	0.15	0.06	0.23	0.13	-0.41	-0.38	0.24	-0.09	0.13	0.24	0.15	-0.36	0.40	-0.17
Co	0.03	-0.05	0.58	-0.29	-0.35	-0.17	-0.13	-0.17	0.10	-0.48	0.01	0.05	-0.28	0.18	-0.10	0.05
Ni	-0.18	0.27	0.36	-0.36	-0.09	0.04	-0.14	0.43	-0.04	0.55	-0.04	0.23	-0.12	-0.22	0.01	0.07
Cu	-0.20	-0.28	0.30	-0.10	0.06	0.16	0.46	-0.42	-0.35	0.31	-0.02	0.16	0.16	0.23	-0.07	-0.17
Zn	-0.31	-0.32	0.07	0.21	0.15	0.14	-0.21	0.13	0.27	0.11	0.30	0.02	0.06	0.30	-0.42	0.44
Se	-0.04	-0.33	-0.14	0.11	-0.51	0.03	-0.39	0.03	-0.48	-0.04	-0.23	0.15	0.26	-0.21	-0.13	0.10

Rb	-0.15	0.44	-0.34	-0.06	0.05	-0.07	-0.07	-0.15	-0.12	-0.13	-0.06	0.65	-0.04	0.38	-0.04	0.13
Sr	0.01	-0.52	-0.20	-0.04	0.04	0.24	0.06	0.30	0.01	-0.07	-0.03	0.30	-0.59	0.06	0.20	-0.22
Mo	0.21	0.05	-0.11	0.17	-0.54	0.22	0.14	-0.30	0.56	0.31	-0.07	0.18	-0.05	-0.06	-0.10	-0.03
Cd	-0.01	-0.27	-0.28	-0.31	-0.13	-0.68	-0.01	-0.11	0.06	0.23	0.44	0.05	0.02	-0.03	0.05	-0.05

NY12	PC1	PC2	PC3	PC4	PC5	PC6	PC7	PC8	PC9	PC10	PC11	PC12	PC13	PC14	PC15	PC16
Standard deviation	1.89	1.42	1.28	1.18	1.05	0.98	0.96	0.94	0.87	0.81	0.78	0.67	0.61	0.53	0.53	0.31
Proportion of Variance	0.22	0.13	0.10	0.09	0.07	0.06	0.06	0.06	0.05	0.04	0.04	0.03	0.02	0.02	0.02	0.01
Cumulative Proportion	0.22	0.35	0.45	0.54	0.61	0.67	0.73	0.78	0.83	0.87	0.91	0.94	0.96	0.98	0.99	1.00
Mg	0.34	-0.16	0.03	-0.21	0.17	-0.29	0.42	-0.16	0.12	0.16	-0.19	0.30	-0.02	-0.47	0.34	0.01
P	0.39	0.02	0.19	0.15	0.06	0.05	0.17	-0.33	0.21	-0.08	-0.16	0.37	0.03	0.53	-0.38	0.03
S	0.31	-0.09	-0.25	-0.20	0.01	0.22	-0.34	0.07	-0.10	-0.44	-0.41	0.03	-0.50	-0.08	0.02	0.02
K	0.14	0.05	0.44	0.35	0.39	0.16	-0.20	0.07	-0.19	-0.37	-0.02	0.05	0.36	-0.10	0.36	0.00
Ca	0.16	0.60	0.05	-0.24	0.07	-0.05	-0.08	0.05	-0.11	0.16	0.03	-0.02	-0.03	0.05	0.05	0.70
Mn	0.37	-0.07	0.10	-0.26	0.10	-0.05	0.24	-0.11	0.04	-0.13	0.04	-0.80	0.10	0.16	0.01	-0.09
Fe	0.37	-0.16	-0.17	-0.04	-0.31	0.02	-0.06	0.24	-0.18	0.15	0.31	0.18	0.04	0.44	0.51	-0.08
Co	-0.01	0.23	-0.16	0.32	-0.07	0.18	0.60	0.56	0.00	-0.08	-0.32	-0.06	-0.02	0.05	0.01	0.00
Ni	-0.02	0.07	-0.41	0.03	0.59	0.08	0.01	0.08	0.49	-0.12	0.41	0.05	-0.12	0.08	0.09	0.00
Cu	0.24	0.02	0.15	0.54	0.15	-0.26	-0.11	0.02	-0.14	0.35	0.11	-0.18	-0.56	-0.06	-0.06	-0.08
Zn	0.36	-0.19	-0.16	-0.05	0.12	0.30	0.06	0.18	-0.36	0.12	0.32	0.10	0.22	-0.32	-0.51	0.06
Se	0.19	0.14	-0.08	0.28	-0.37	0.54	-0.08	-0.32	0.39	0.17	0.02	-0.15	0.07	-0.29	0.16	0.05
Rb	0.04	-0.23	0.37	-0.23	0.15	0.20	-0.25	0.47	0.39	0.44	-0.25	-0.02	0.02	0.07	-0.05	-0.02
Sr	0.11	0.63	0.02	-0.24	0.07	0.05	-0.09	0.00	-0.09	0.13	-0.02	0.11	0.03	-0.05	-0.03	-0.69
Mo	0.25	0.11	0.14	0.05	-0.38	-0.43	-0.16	0.33	0.39	-0.37	0.22	0.05	0.08	-0.23	-0.19	-0.01
Cd	0.14	-0.01	-0.51	0.23	0.11	-0.34	-0.31	-0.01	0.02	0.19	-0.41	-0.12	0.47	0.04	-0.04	0.01

MO06	PC1	PC2	PC3	PC4	PC5	PC6	PC7	PC8	PC9	PC10	PC11	PC12	PC13	PC14	PC15	PC16
Standard deviation	2.01	1.48	1.30	1.20	1.11	1.04	0.89	0.78	0.77	0.74	0.70	0.67	0.58	0.49	0.37	0.33
Proportion of Variance	0.25	0.14	0.10	0.09	0.08	0.07	0.05	0.04	0.04	0.03	0.03	0.03	0.02	0.02	0.01	0.01
Cumulative Proportion	0.25	0.39	0.50	0.59	0.66	0.73	0.78	0.82	0.86	0.89	0.92	0.95	0.97	0.98	0.99	1.00
Mg	-0.26	0.12	-0.02	0.05	-0.25	0.54	-0.28	0.08	-0.16	0.09	-0.60	0.12	-0.24	-0.01	0.00	-0.08
P	-0.41	0.06	-0.09	0.04	0.01	0.24	0.33	0.05	0.01	-0.15	0.03	0.05	0.37	0.35	-0.43	0.42
S	-0.30	0.26	-0.05	0.16	0.25	-0.29	0.16	-0.13	-0.15	-0.48	-0.08	-0.01	-0.53	0.22	0.21	0.03
K	-0.38	0.03	0.15	0.12	-0.05	0.11	0.52	-0.09	0.15	-0.04	-0.05	-0.03	0.25	-0.47	0.28	-0.37
Ca	-0.16	-0.51	0.15	0.07	0.34	0.06	0.00	0.08	-0.20	0.01	0.12	0.21	-0.18	0.08	-0.42	-0.48
Mn	-0.22	0.10	-0.24	0.45	0.04	0.29	-0.29	-0.01	-0.24	0.16	0.56	-0.20	0.06	0.04	0.25	-0.05

Fe	-0.33	0.11	0.13	-0.22	-0.02	-0.35	0.04	-0.04	-0.18	0.61	-0.03	0.26	0.11	0.37	0.25	-0.07
Co	-0.11	-0.27	-0.44	0.13	-0.12	-0.31	0.22	0.49	-0.20	0.23	-0.18	-0.29	-0.15	-0.22	-0.06	0.15
Ni	-0.08	-0.29	-0.52	-0.25	0.17	-0.04	-0.23	-0.13	0.00	-0.29	-0.24	-0.06	0.42	0.18	0.26	-0.23
Cu	-0.22	-0.07	-0.01	-0.52	-0.25	0.17	0.00	0.45	0.28	-0.18	0.38	0.02	-0.26	0.11	0.18	-0.08
Zn	-0.24	0.28	-0.35	-0.32	0.14	-0.11	-0.20	-0.19	-0.04	0.05	0.16	0.32	-0.07	-0.54	-0.30	0.09
Se	-0.21	0.17	0.18	0.35	-0.01	-0.36	-0.43	0.45	0.32	-0.18	-0.08	0.17	0.26	-0.04	-0.09	-0.10
Rb	-0.30	-0.22	-0.06	0.10	-0.26	-0.15	-0.15	-0.47	0.52	0.17	-0.01	-0.33	-0.22	0.10	-0.21	-0.03
Sr	-0.19	-0.52	0.24	0.06	0.17	0.05	-0.16	-0.07	0.04	-0.03	-0.02	0.24	-0.04	-0.22	0.35	0.58
Mo	-0.22	0.02	0.43	-0.31	-0.06	-0.12	-0.23	-0.03	-0.44	-0.16	0.00	-0.56	0.15	-0.15	-0.13	0.04
Cd	-0.04	0.19	0.04	-0.14	0.72	0.20	-0.02	0.19	0.33	0.29	-0.15	-0.35	-0.06	0.00	0.03	0.05

SA10	PC1	PC2	PC3	PC4	PC5	PC6	PC7	PC8	PC9	PC10	PC11	PC12	PC13	PC14	PC15	PC16
Standard deviation	2.37	1.57	1.19	1.07	1.01	0.93	0.83	0.75	0.71	0.69	0.60	0.54	0.46	0.42	0.35	0.28
Proportion of Variance	0.35	0.15	0.09	0.07	0.06	0.05	0.04	0.04	0.03	0.03	0.02	0.02	0.01	0.01	0.01	0.00
Cumulative Proportion	0.35	0.51	0.59	0.67	0.73	0.78	0.83	0.86	0.89	0.92	0.95	0.96	0.98	0.99	1.00	1.00
Mg	0.31	-0.12	0.24	-0.17	0.39	-0.07	0.12	-0.32	0.20	-0.09	0.16	-0.16	0.14	-0.38	-0.34	-0.39
P	0.32	-0.21	0.31	0.12	0.07	0.11	-0.18	-0.27	0.08	0.11	0.03	0.05	0.17	0.00	0.75	0.10
S	0.29	-0.19	0.15	-0.12	0.09	-0.15	-0.27	0.58	0.09	-0.31	-0.22	0.43	0.01	-0.23	-0.06	0.11
K	0.24	-0.12	0.36	0.15	-0.49	0.09	0.11	-0.03	-0.05	-0.31	-0.32	-0.50	-0.04	0.02	-0.18	0.18
Ca	0.13	0.49	0.23	-0.06	0.12	-0.16	0.29	-0.04	-0.19	-0.11	-0.27	0.13	-0.54	-0.01	0.26	-0.26
Mn	0.36	0.15	0.07	-0.09	0.18	0.18	0.06	-0.23	0.13	0.29	-0.02	0.23	-0.20	0.20	-0.34	0.60
Fe	0.30	0.09	-0.08	-0.34	-0.01	-0.31	-0.01	0.25	-0.22	-0.07	0.58	-0.38	-0.10	0.07	0.15	0.21
Co	0.26	0.20	-0.37	-0.12	-0.20	-0.02	-0.22	0.10	0.27	0.47	-0.32	-0.27	-0.07	-0.38	0.09	-0.09
Ni	0.16	0.28	-0.23	0.36	0.17	0.06	-0.65	-0.25	-0.26	-0.34	0.03	-0.07	-0.07	-0.01	-0.12	-0.03
Cu	0.29	-0.13	-0.08	0.10	-0.13	0.33	0.20	0.09	-0.70	0.25	0.07	0.21	0.12	-0.26	-0.09	-0.14
Zn	0.36	-0.19	-0.12	-0.04	0.10	0.02	-0.06	0.15	0.03	0.12	-0.17	-0.07	0.03	0.73	-0.08	-0.45
Se	-0.11	0.29	0.33	0.03	0.17	0.66	-0.10	0.43	0.16	0.05	0.21	-0.22	-0.04	0.00	0.01	-0.10
Rb	0.25	0.19	-0.04	0.21	-0.56	0.02	0.06	-0.06	0.37	-0.11	0.44	0.37	-0.05	0.02	-0.05	-0.23
Sr	0.08	0.56	0.07	-0.10	-0.01	-0.12	0.12	0.03	-0.06	-0.06	-0.16	0.05	0.76	0.11	0.00	0.06
Mo	-0.17	0.05	0.55	0.00	-0.20	-0.35	-0.42	-0.01	-0.18	0.46	0.08	0.06	-0.02	0.06	-0.21	-0.13
Cd	0.10	0.01	0.00	0.76	0.27	-0.33	0.25	0.27	0.10	0.20	0.07	-0.14	0.03	-0.05	-0.03	0.10
