# Supplementary material for: Multivariate analysis reveals environmental and genetic determinants of element covariation in the maize grain ionome
Source: Plant Direct. 2019 May 10;3(5):e00139. doi: 10.1002/pld3.139 (PMC6589523; doi:10.1002/pld3.139)
Supplement: Supplementary file 8 [file PLD3-3-e00139-s008.pdf]

Location	Weather Station
Florida	Homestead General Aviation Airport
Indiana	West Lafayette 6 NW
North Carolina	Clayton Field
New York	Aurora Research Farm
Missouri	Columbia U of M

Table S4. Weather Station Locations. Location and name of weather station from which weather data was obtained.
