# Supplementary material for: Multivariate analysis reveals environmental and genetic determinants of element covariation in the maize grain ionome
Source: Plant Direct. 2019 May 10;3(5):e00139. doi: 10.1002/pld3.139 (PMC6589523; doi:10.1002/pld3.139)
Supplement: Supplementary file 9 [file PLD3-3-e00139-s009.pdf]

REVIEWER COMMENTS:

Reviewer: 1

TPJ-00891-2018 is an ambitious manuscript that treats questions in both plant physiology and statistical genetics using a large-scale data set and a series of multi-variate approaches. Clearly, much effort and craft have gone into the analysis, construction of arguments, and written presentation. However, the complexities of both the topics and methods combined with data set weaknesses render the manuscript inaccessible; as a reader, I have found it difficult to reach clear and memorable conclusions even after considerable study of the manuscript. However, based on the apparent unmasking of interesting (even if unsurprising) phenomenologies, I fundamentally agree that valuable information can be gleaned from these data using complex approaches. I'm just not convinced that the focus on PCs and aPCs is very useful in its present form.

I present below two lists of points to consider. The first list is technical and is aimed at meeting what I view as the requirements for publication. The second list contains suggestions to make the manuscript more accessible and more convincing. Neither list is provided in any particular order of importance.

Technical issues:

• Fig 4a has both logical and arithmetic flaws: what is the basis for non-reciprocal overlap in this quasi-venn? The text is not clear, and overlap is not defined. Also, 180 is not equal to 172.
The reviewer is correct that 180 in the diagram's title is a mistake, and we have corrected it to 172 to reflect the sum of the diagram's components (172). This number is listed correctly in the figure legend. The legend for Figure 4a goes into detail defining non-overlapping and overlapping regions of the diagram.

• Figure 4c and Table S2 work together to provide an insufficiently clear picture of some of the most important results. Fig 4c is much too coarse-- "QTL are represented as dashes of uniform size for visibility" is not acceptable for a manuscript that wishes to illuminate circumstances surrounding hypotheses of pleiotropy. A good figure with accuracy and precision is needed to be convincing. Why not show one of the featured regions with adequate detail such as what is shown in Fig 2? The remaining useful visualizations could then be placed in a supplementary figure.
In order to show results visibly, we needed to use uniform sized dashes, which is stated in the caption. We do not make any statements about the window size of QTL in relation to this plot. Panel B shows a region in blown-up detail to emphasize the discussion around this figure which centers around the result that the PC approach detects both previously found and new QTL. In order to show one of the featured regions with adequate detail, we created a new figure (now figure 5) that shows the chromosome 7 featured region in detail.

Table S2 is still missing the estimates for effect and R-square. I recognize for PCs as traits, units are hard to interpret. However, effect direction and relative magnitude remain relevant, especially for linked loci of the same PC trait, such as for the example highlighted in Fig 4b. R-squares are needed too—how much variance is explained by these loci? Also, please define "MaxPerm" in caption for Table S2.
The term "MaxPerm" has been changed "LOD Threshold" and described in the caption. Effect size, direction, and percent variance explained by each QTL have been added to the table.

• In Fig 6a, Q1-4 is missing for GDD and Q4 label is also missing.
This has been fixed. Q1-4 was not missing for GDD, but the Q4 label was. We added lines to delineate sections to make it easier to visually separate the table for different traits.

• In Fig 6a and 6d, there is no presentation of p values and no correction for multiple tests.
We make it clear in our results and discussion that the weather data is very limited and that we cannot make any definitive conclusions based on this weather data and the correlations with only 9 environments.

• Color legend is missing from Fig S4.
We added a color legend to figure S4.

• Did GDD calculation not have min and max thresholds? Please clarify either way.
No. We added this detail to the manuscript.

• Fig 6 legend should indicate that soil and weather variables are indirect to varying degrees and point readers to the M&M section or to a more complete Table S4. In-field in-season quantifications are quite feasible and are often used, so the use of indirect data should be made clear. As such, Table S4 should report the distance between each field used and the location of the associated weather station. Likewise, if WSS reports the year of the survey used, Table S4 could also report that relative to the year of the IBMRIL seed production.
We do not know the exact field location at each field site for all of the growouts. The weather variables reflect location averages over time. As we discuss in the paper, this weather data cannot provide solid evidence for PC-weather variable relationships, however, our approach could be applied to future work with more detailed environmental data collection.

• The introduction is great in general, but the choice of citations makes the PCA approach seem newer than it is. Please cite these respective plant and animal examples from 2006 and 2005: https://link.springer.com/article/10.1007/s00122-006-0359-2 and https://doi.org/10.2527/2005.83112471x, or studies pre-dating these.
At least 9 sources were cited in the introduction in relation to PCA, chosen based on relevance to this particular study. We included a 2009 review by Baxter et al. which includes information on earlier studies.

• Tables 1 and 2 contain incorrect usages of the term QTL in the context of pleiotropic hypotheses. These are cases where the independent QTLs for multiple traits are co-located. While an underlying polymorphism may exert effects on multiple traits, a QTL, by definition, is associated only with the phenotypic trait used to detect it. Please review all usage instances and rephrase in cases that need it.
The titles for tables 1 and 2 have been changed and the usage of the term QTL in the corresponding text has been checked and rephrased where necessary to reflect the 1-1 relationship of a single quantitative trait locus and a single trait.

Strategic issues:

• I think the manuscript would be strengthened if it were less focused on the methods used. To make it more focused on the biology, specific hypotheses should be tested. At present, lines 70-74 set up the study for weak success by setting the bar too low. Why not use the correlation data and network approaches to make hypotheses about "the exact nature of these relationships" (line 70) and then test them?
We do not know the exact nature of all element-element relationships, which also vary by environment, so using an undirected multivariate approach allows the actual data collected from each environment (which reflects elements that co-vary) to reveal patterns about the relationships.

• To the degree that the current manuscript focuses on methods, I question the quality of the experimental design. Is there any biological replication within each trial and if so, is it balanced and usable? If not, what are the implications for the use of multi-variate methods in distinguishing among competing explanations? On this topic, lines 394-410 would benefit from citations of statistical methods and from a more thorough treatment. Critically, L396-398 is a statement that could not reasonably be evaluated by most readers of TPJ. I recommend using smaller chunks of data to make important points more concretely. Figure 1 is a great example of this. The investment in methods to fix experimental design weaknesses creates distractions and injects uncertainty.
Replication was described in different field trials, which have varying levels of replication, in our previous publication with this data (Asaro et al., 2016, G3). The description of the PCA projections here is given to reflect our usage of PCA projections in the paper (to test against the aPCs that were actually used in further analyses and provide a discussion of a method that could be applied in future studies). Our methods descriptions are detailed to a level appropriate for a "sound science" paper.

• In setting the stage for the importance of leveraging covariance, lines 2-5 should be rephrased and augmented. Would the statements be less true if rearranged? I think that pleiotropy, E, GxE, epistasis, and circumstantial epistasis are the underpinnings of, rather than the results of the integrated responses. The omission of all GxG considerations (epistasis, and circumstantial epistasis) from this list is flawed, especially since much of the reduction in data dimensionality by PCA comes from using phenotypic circumstance, rather

than the underlying genotypic data at a myriad of loci. By extension, searches for epistatic interactions within the genetic network may be a very useful approach.
In the introduction, we have pointed out the role of genetic regulation of multiple elements which includes examples of pleiotropy and epistasis. We go into detail about the fact that the multivariate approach has been developed to potentially uncover genes that regulate multiple traits in both the introduction and discussion. While we did not formally test GxG interactions, we are not discounting the importance and presence of such interactions, along with GxE interactions, in determining the ionome.

• Fig 5b is redundant with 5a. I suggest removing 5b. Also, it is a bit unclear how Fig 5a and Fig S4 are different beyond the fact that one includes loadings and that Fig S4 reports aPCs, while 5a does not. Why not show results from Figs 5a and S4 together of just Fig S4, depending on the points being made?
We do not discuss in detail the specific element loadings on aPCs in the manuscript so the figure with overlaid element loadings is more appropriate for the supplemental information while the simpler figure gets across our main point that the PCs separate out environments without distracting the reader.

• Figure 3 could be a supplement.

• Figure 7b needs the same improvements as suggested above for Fig 4c.
The discussion of these results does not focus on any particular QTL in detail but rather on the general concept of PCs having both environmental and genetic underpinnings, so a blown up inset of a specific QTL region would not be relevant in this figure, but we did create an additional figure (figure 5) to show in detail a region highlighted in figure 4.

• Homeostasis is invoked often in the manuscript, but never discussed in the physiological context of the whole grain that has been ground to form the sample. The dry and dormant nature and the concept of sequestration are overlooked.
We don't believe this discussion is necessary nor appropriate for this paper.

• In line 350, what are the homeostatic mechanisms and concurrent multi-element behaviors? How are these different, and could citations be given for each to allow more formal learning by the reader.
Citations are included on lines 347-349 that describe homeostatic mechanisms between Fe and Zn and Mn and Mo.

Reviewer: 2

Ref: TPJ-00891-2018
Title: Multivariate analysis reveals environmental and genetic determinants of element covariation in the maize grain ionome
Journal: the plant journal

Authors in this manuscript did principle component analysis (PCA) intensively to explore the QTLs with pleiotropy effects on multi-elements. They summarized data from single elements in previous publication and did element-to-element traits correlations. Further, authors analyzed PCs within environment and across environments. Based on the PCs, QTL were detected and compared with single element QTL detection results. Authors found novel loci, and thought these loci were controlling covariance between/among elements. In the analysis of across environment PC, lines in same environments were clustered together, the first several PCs were thought as environmental variables, and were correlated with environmental data, such as temperature and soil.

Although the topic of element covariation responsive to genetic and environmental variation is interesting, this research seems dry on analysis, less on biology. The analyses in this manuscript can be summarized to three: correlation, PCA, and QTL mapping. I'm not critical to the analysis, but it will be much better if the statistical results could be proved in biology pathways. Authors mentioned two hypothesis on covariance: genes responsible to the transport and genes sharing homeostatic pathways. It should be clear that these two hypothesis were supported by QTL results from PCA.

As we know, QTL detected for single element involved genes responsible to the corresponding element. QTL detected for PC involved genes responsible to covariance. In order to make sure how many and which elements caused this covariance, we need to check the loading values and also check the coincidence with QTL in single element (Table 2). The logic here seems confusing, because first, they conducted QTL detection for single element; second, conducted QTL detection for PC; third, went back to compare with QTL for single element.

Using PCs as traits to detect QTL has been studies in GWAS and Linkage mapping. Even they did find novel loci, however, trying to explain the novel loci and the corresponding PC is not that easy. In this study, if authors would like to dedicate themselves to explain these novel loci and the major PCs, putting these novel loci in ionome pathways, and supporting one of covariance hypothesis, the novelty of this manuscript will show up.

Some minor comments are as follows:

1) In the across environments PCA, authors stacked lines in all the environments together, and found PC1 and PC2 separate these lines based on environments. The original input matrix for PCA should be No. of lines × No. of environment row and 16 columns. What if the original input matrix is No. of lines row and No. of environment × 16 columns? The number of observation is No. of lines and the number of variable is No. of environment ×

No. of single elements columns? Is this analysis combining all the environments and showing novel results?
The analysis combining all environments allows us to look at the effect of environment. The within-environment analysis allows us to use the multi-variate approach without adding in GxE, while the across-environment approach produces traits that can be examined in the context of GxE. Both approaches facilitate detection of QTL that were not detected using single element traits.

2) Positions of QTL were studied very well, and compared using different methods. However, effect of QTL is less mentioned. Authors explained that QTL effects are not accurate because of Beavis effect or small population size. The effect in small population size is overestimated to some extent, but the direction of effect may provide information for the positive and negative relationships between elements.
The loadings of each PC itself provides information for the positive and negative relationships between the elements that go into determining the PC. We have added effect magnitude and direction of each PC QTL to table S2.

3) In fig.4, authors provided QTL mapping results for PC5 in one location and attempted to support the novel loci detected for PC5. Also, fig. 4C provided QTL positions in each environment. From these results, I try to think that why QTLs were detected for PC1, not PC2, or why was PC5, not PC6. I don't have clue to figure out that.
Slightly different combinations of elements in each PC lead to the distinct results. Biological interpretation beyond this is difficult and was not a goal. The motivation was to find interesting loci that may be related to adaptive traits rather than understand the actual trait meaning of the PC traits.

Also, in fig.7, authors listed number of QTL detected for each PC. There is still no clue to figure out why PC4 and PC5 could detect the most number of QTL.
The simplest explanation is that these PCs had the most genetic variance associated with them. Each PC differs based on the loadings of different elements into each PC. PC1-3 may have more artifact or uncontrolled environmental variation loading into them. One advantage of this approach is that non-genetic factors can segregate into a small number of PCs, leaving more genetic variation to be accounted for by other PCs.

All these questions went back to my major concern: how to explain these results? What are the biological meaning of these results?
Biological interpretation is difficult and was not the goal. We were interested in how novel approaches to the data might alter the interpretation of elemental traits. The motivation was to find interesting loci that may be related to adaptive traits rather than understand the actual trait meaning of the PC traits.
